# Isolation, Total Synthesis and Anti-Diabetic Activity of Filiforidine from *Cassytha filiformis*

**DOI:** 10.3390/molecules30244763

**Published:** 2025-12-12

**Authors:** Caiyun Zhang, Hong Zhu, Fang Zhang, Yuexia Jiang, Zibao Huang, Dong Lin, Niangen Chen, Xiaopo Zhang, Yanhui Fu

**Affiliations:** 1 College of Chemistry and Chemical Engineering, Hainan Normal University, Haikou 571158, China; hy0308018@muhn.edu.cn; 2 School of Pharmaceutical Science, Hainan Medical University, Haikou 571199, China; 18789928157@163.com (H.Z.); hy0207102@muhn.edu.cn (F.Z.); hy0207025@muhn.edu.cn (Y.J.); huangzibao0107@163.com (Z.H.); donglin793@126.com (D.L.)

**Keywords:** *Cassytha filiformis*, Filiforidine, total synthesis, diabetes

## Abstract

*Cassytha filiformis* is a folkloric herbal medicine used to treat type 2 diabetes mellitus (T2DM). In this study, an oxidized aporphine alkaloid, designated as Filiforidine (3,10,11-trimethoxy-1,2-methylenedioxy-7-oxoaporphine), was isolated from *C. filiformis*, and its structure was elucidated through comprehensive spectroscopic analysis. Owing to its novel structure and significant glucose consumption activity, the total synthesis of Filiforidine was achieved for the first time. The key steps featured an electrophilic addition reaction, involving the reduction of a nitro group to an amino group with lithium tetrahydroaluminum, and a copper bromide-catalyzed oxidative aromatization reaction as well as a photocyclization reaction. Several experimental steps were optimized. Furthermore, a complex post-treatment method was developed, which reduced the column chromatography separation steps. Specifically, 2-(4-methoxybenzo[d][1,3]dioxol -5-yl) ethan-1-amine is salted with dilute hydrochloric acid. Cytotoxicity assay and glucose oxidase assay showed that Filiforidine had significant glucose consumption-promoting effects on HL-7702 cells at 0.625 μM, 1.25 μM, and 2.5 μM but without cytotoxicity. Therefore, Filiforidine might be a promising drug candidate for the treatment of diabetes.

## 1. Introduction

Type 2 diabetes primarily results from genetic and environmental factors that lead to insulin resistance and impaired insulin secretion in peripheral tissues, resulting in a relative or absolute deficiency of insulin in the body, which reduces glucose uptake and utilization, thus triggering hyperglycemia and leading to diabetes [1]. About 589 million adults worldwide had diabetes in 2025, according to data from the International Diabetes Federation. It was postulated that if adequate measures are not taken, this number will increase exponentially to around 853 million by 2050 [2,3]. Diabetes has become a major public health concern that poses a serious threat to human health [4]. Glucose-lowering agents such as sulfonylureas, biguanides and α-glucosidase inhibitors are widely used in clinic for treating diabetes, but there are side effects or toxics such as reduced efficacy and poor glycemic control after long-term use, as well as potential adverse effects [5]. Therefore, the development of new anti-diabetic drugs that are safer and more effective, with better patient compliance, remains a major focus of current research.

Studies have shown that natural products are an important source for the development of new drugs for the treatment of diabetes. Traditional Chinese medicine (TCM) has a long history of preventing and treating diabetes, and is characterized by precise efficacy, moderate effects and low toxicity. TCM extracts such as *Morus alba*, Perilla leaf and *Momordica charantia*, are effective in the treatment of T2DM. [6,7,8]. According to statistics, between 1981 and 2019, 1394 small molecule drugs were approved for marketing worldwide, 64.1% of which were directly or indirectly derived from natural products, of which 35 out of 39 anti-diabetic small molecule drugs were derived from natural products. In particular, the nine anti-diabetic drugs approved from 2015 to 2019 were derived from natural origin [9]. In March 2020, China’s first original natural anti-diabetic drug, Mulberry Twig Alkaloids Tablets, was approved for marketing [10]. *Cassytha filiformis* (Lauraceae), a parasitic herb, is widely distributed in tropical and subtropical regions worldwide. It mainly grows in Hainan, Guangxi, Guizhou provinces of China. It is used as a TCM for improving the situation of diabetes, tumors and parasites. Pharmacological investigations indicated that the extracts of *C. filiformis* possessed anti-diabetic, anti-tumor and anti-parasitic activities [11,12,13]. For example, the methanolic extract of *C. filiformis* was able to reduce the blood glucose by 46.8% using alloxan induced diabetic mice [14]. Chemical studies on this species have resulted in the isolation of alkaloids, flavonoids, volatile oils, and the aporphine alkaloids have been assigned as its characteristic components [15]. Notably, their intriguing chemical structures and relevant biological activities attracted our interests. Previous studies have also reported the anti-diabetic effects of various aporphine alkaloids [16,17]. Based on these, the present study aimed to isolate and characterize the potential anti-diabetic components from the TCM of *C*. *filiformis* and then to achieve the first total synthesis of the active compound, Filiforidine, to unambiguously confirm its structure and ensure a reliable supply for biological evaluation.

## 2. Results and Discussion

In our ongoing search for anti-diabetic natural products from *C. filiformis*, bioassay-guided fractionation of the ethanol extract, followed by acid–base extraction and multiple chromatographic purifications, led to the isolation of 9.1 mg of Filiforidine from 50 kg of dried plant material. Due to the minute quantity obtained, which was insufficient for comprehensive biological evaluation and structural verification, we subsequently undertook its total synthesis. The following sections described the detailed structural elucidation and the first total synthesis of Filiforidine.

### 2.1. Extraction and Identification of Filiforidine

**Figure 1:**
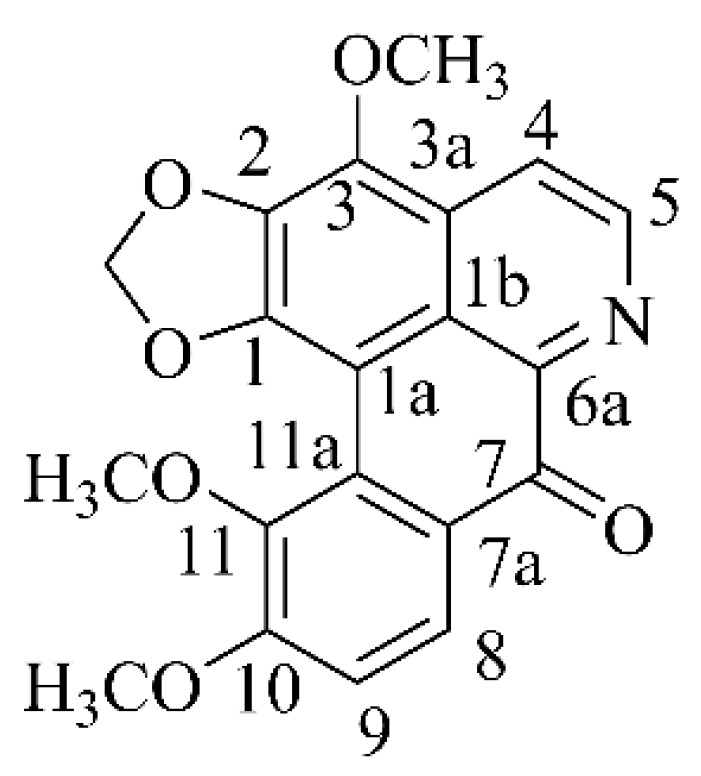
Structure of Filiforidine.

**Figure 2:**
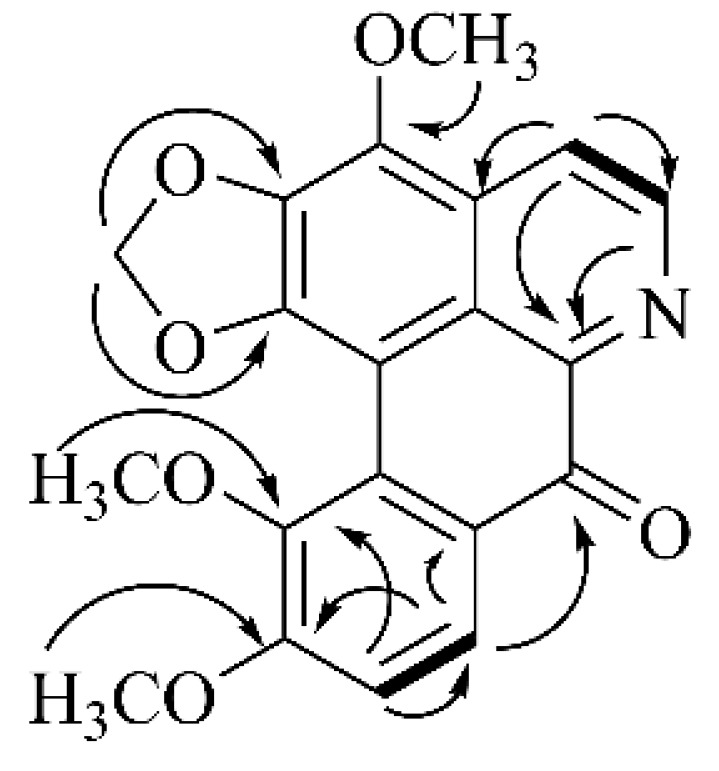
^1^H-^1^HCOSY (thick line) and HMBC (arrow) correlations of Filiforidine.

Filiforidine (9.1 mg) was obtained as a yellow amorphous powder and showed positive reaction with Dragendorff’s reagent. Its molecular formula (C_20_H_15_NO_6_) was determined by HRESIMS at *m*/*z* 366.0980 [M + H]^+^ (calculated for 366.0978). Its UV spectrum showed absorptions at 218.4 (3.60), 256.2 (3.67), 285.4 (3.72), and 359.4 (3.82) nm. The IR spectrum exhibited absorptions at 3442 (N-H), 1646 (C=O), 1587, 1449 (C=C), 1022 (C-N) cm^−1^. The ^1^H NMR data displayed signals for three methoxy groups [*δ* = 3.80 (3H, s, 11-OCH_3_), 3.96 (3H, s, 10-OCH_3_), 4.21 (3H, s, 3-OCH_3_)], a methylenedioxy group [*δ* = 6.36 (2H, s, -O-CH_2_-O-)]. In the aromatic region, a pair of AB doublets at *δ* 8.76 (1H, d, *J* = 5.2 Hz, H-4), 8.10 (1H, d, *J* = 5.2 Hz, H-5) were characteristic signals of H-4 and H-5 of oxoaporphine derivatives [18]. Another pair of AB doublets at [*δ* = 8.07 (1H, d, *J* = 8.8 Hz, H-8), 7.34 (1H, d, *J* = 8.8 Hz, H-9)] were assigned to H-8 and H-9. The ^13^C NMR spectrum of the compound resolved twenty carbon resonances attributable to one ketone group [*δ* = 180.9 (C-7)], a methylenedioxy group [*δ* = 103.1 (-O-CH_2_-O-)], a quinoline group [*δ* = 150.4 (C-1), 138.6 (C-2), 135.8 (C-3), 144.5 (C-4), 118.6 (C-5), 99.8 (C-1a), 123.2 (C-1b), 130.5 (C-3a), 144.2 (C-6a)], a benzene ring [*δ* = 126.5 (C-7a), 125.3 (C-8), 113.2 (C-9), 158.3 (C-10), 146.0 (C-11), 126.2 (C-11a)], three methoxy groups [*δ* = 61.0 (11-OCH_3_), 60.8 (3-OCH_3_), 56.8 (10-OCH_3_)]. The NMR spectral data, which displayed signals characteristic of a quinoline moiety bearing a C-7 ketone group, revealed the structural features of an oxoaporphine alkaloid, as shown in Figure 1. Its ^1^H-^1^H COSY spectrum revealed the linkage of C_4_–C_5_ and C_8_–C_9_ from correlations between H-5 (*δ*_H_ 8.10) and H-4 (*δ*_H_ 8.76), H-9 (*δ*_H_ 7.34) and H-8 (*δ*_H_ 8.07) as shown in Figure 2. The HMBC spectrum (Figure 2) showed correlations of 10-OCH_3_ (*δ*_H_ 3.96) to C-10 (*δ*_C_ 158.3), 11-OCH_3_ (*δ*_H_ 3.80) to C-11 (*δ*_C_ 146.0), H-9 (*δ*_H_ 7.34) to C-8 (*δ*_C_ 125.3) and C-11 (*δ*_C_ 146.0), and correlations of H-8 (*δ*_H_ 8.07) to C-7 (*δ*_C_ 180.9) confirmed that 10-OCH_3_ and 11-OCH_3_. In addition, the correlations of H-4 (*δ*_H_ 8.76) to C-5 (*δ*_C_ 118.6), C-3a (*δ*_C_ 130.5) confirmed that it possessed the same carbon skeleton as isofiliformine. Further checking the NMR data and comparing it with literature, it was similar to isofiliformine, except that the 9-OH was replaced by 11-OCH_3_ [18]. After carefully checking the literature, the compound has been reported in 2021 from *Fissistigma oldhamii* var. *Longistipitatum* [19]. Thus, it was assigned as 3,10,11-trimethoxy-1,2-methylenedioxy-7-oxoaporphine. The complete data of ^1^H NMR and ^13^C NMR were depicted in Table 1.

### 2.2. Total Synthesis of Filiforidine

**Figure 3:**
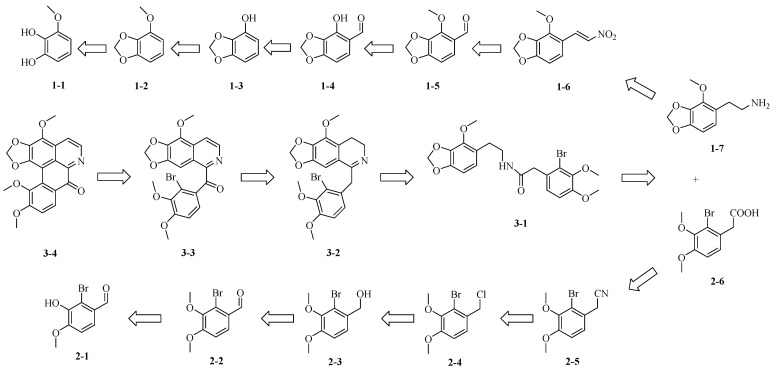
Retrosynthetic analysis of Filiforidine.

The remarkably low natural abundance of Filiforidine in *C. filiformis* presented a significant bottleneck to a comprehensive investigation of its potential therapy. To overcome this limitation and ensure a reliable supply for rigorous biological evaluation, the first total synthesis of Filiforidine was accomplished. The synthetic route of Filiforidine employed a convergent approach, utilizing 3-methoxycatechol as the starting material to prepare 2-(4-methoxybenzo[d][1,3]dioxol-5-yl)ethan-1-amine (**1-7**), and 2-bromo-3-hydroxy-4-methoxybenzaldehyde as the starting material to synthesize 2-(2-bromo-3,4-dimethoxyphenyl)acetic acid (**2-6**). Subsequently, Compounds **1-7** and **2-6** were coupled to generate the key intermediate 2-(2-bromo-3,4-dimethoxyphenyl)-N-(2-(4-methoxybenzo[d][1,3]dioxol-5-yl)ethyl)acetamide (**3-1**). The retrosynthetic synthetic route, as delineated in Figure 3, features key steps including a copper bromide-catalyzed oxidative aromatization and a photocyclization reaction, providing efficient access to the oxidized aporphine scaffold.

The synthesis steps of Filiforidine commenced with (2-bromo-3,4-dimethoxyphenyl) (9-methoxy-[1,3]dioxolo [4,5-g]isoquinolin-5-yl) methanone (**3-3**), and a debromination ring-closing reaction were performed using sodium borohydride-catalyzed photocyclization. Moreover, **3-3** was obtained from 5-(2-bromo-3,4-dimethoxyphenyl)-9-methoxy-7,8-dihydro-[1,3] dioxolo [4,5-g] isoquinoline (**3-2**) through the oxidation and aromatization reactions catalyzed by copper bromide and DBU. Cyclized imine **3-2** was synthesized from 2-(2-bromo-3,4-dimethoxyphenyl)-N-(2-(4-methoxybenzo[d][1,3]dioxol-5-yl) ethyl) acetamide (**3-1**) by means of the Bischler-Napieralski reaction under acidic condition. Ultimately, amide **3-1** was obtained by coupling 2-(4-methoxybenzo[d][1,3]dioxol-5-yl) ethan-1-amine (**1-7**) with 2-(2-bromo-3,4-dimethoxyphenyl) acetic acid (**2-6**) under standard peptide coupling conditions.

Compounds **1-7** and **2-6** were synthesized via two distinct synthetic routes. Compound **1-7** was prepared by reducing the nitro groups in Compound **1-6**. Compound **1-6** was synthesized from Compound **1-5** via a Henry reaction and Compound **1-5** was obtained via the methylation of Compound **1-4**. Compound **1-4** was formed by introducing an ortho-aldehyde group into Compound **1-3**, while Compound **1-3** was derived from Compound **1-2** through demethylation. Compound **1-2** was obtained by the ring closure of Compound **1-1**. As for the other reaction route, Compound **2-6** was generated by hydrolyzing the cyano group in Compounds **2-5**, and **2-5** was obtained by the cyanation of Compound **2-4**. Compound **2-4** was produced by a halogen substitution reaction of Compound **2-3**, and **2-3** was prepared by a reduction reaction of the aldehyde group in Compound **2-2**. Finally, Compound **2-2** was generated through a methylation reaction of Compound **2-1** (Figure 3).

**Figure 4:**
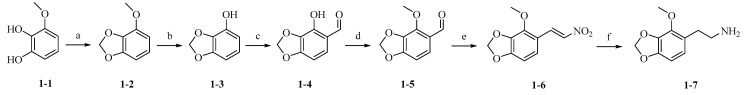
Conditions: (a) CH_2_I_2_, K_2_CO_3_, DMF, 150–160 °C, 75.0%; (b) C_3_H_9_ClSi, NaI, CH_3_CN, 0–82 °C, 87.6%; (c) PFA, MgCl_2_, TEA, 65–82 °C, 78.0%; (d) NaH, CH_3_I, DMF, 20–30 °C, 82.0%; (e) CH_3_NO_2_, NH_4_OAc, HOAc, 118 °C, 87.0%; (f) LiAlH_4_, THF, 0–30 °C, 73.3%.

The starting material (Figure 4) was the commercially available compound of 3-methoxycatechol. Due to the presence of two phenolic hydroxyl groups, the compound was chemically active and likely to participate in subsequent reactions. Hence, it was essential to firstly carry out a ring closure to obtain a relatively more stable methylenedioxy structure. Through the comparison of various conditions and based on the reaction status, appropriate reaction conditions were determined as diiodomethane (two equivalents), potassium carbonate (three equivalents), with DMF serving as the solvent, and refluxing at 150–160 °C, thereby obtaining Compound **1-2** with a yield of 75.0% [20]. Subsequently, Compound **1-2** underwent a demethylation reaction, which facilitated the introduction of an aldehyde group at the adjacent position in the subsequent step and reduced the occurrence of by-products at other positions. The reaction conditions involved using trimethylchlorosilane (three equivalents relative to Compound **1-2**) and sodium iodide (four equivalents relative to Compound **1-2**) in acetonitrile under reflux at 82 °C [21]. The obtained Compound **1-4** had an aldehyde group introduced at the adjacent position under the following reaction conditions of anhydrous magnesium chloride (1.5 equivalents), PFA (4 equivalents, added in two portions), triethylamine (2 equivalents), with acetonitrile as the solvent. The temperature was initially maintained at 65 °C, and then was raised to 82 °C for reflux [22]. After the introduction of the adjacent aldehyde group, the phenolic hydroxyl group at the **C-3** remained chemically active and could lead to participation in subsequent reactions to generate by-products. The target compound required a methoxy group at this position; in this step, the **C-3** was methylated to obtain Compound **1-5**, which has relatively stable chemical properties. The reaction conditions were sodium hydride (2 equivalents), iodomethane (1.3 equivalents), with DMF as the solvents, and the reaction was conducted at room temperature [23]. Subsequently, Compound **1-5** underwent a Henry reaction under the conditions of nitromethane (4 equivalents), ammonium acetate (1.3 equivalents), with acetic acid as the solvent, and refluxing at 118 °C [24,25,26,27]. Finally, the strong reductant lithium aluminum hydride was used to transfer Compound **1-6** into **1-7**, and the reaction conditions were lithium aluminum hydride (4 equivalents), temperature range of −5 °C to 30 °C, and dry tetrahydrofuran as the solvent [28,29] (Figure 4).

**Figure 5:**
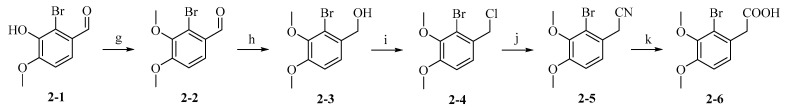
Conditions: (g) NaH, CH_3_I, DMF, 0–10 °C, 86.0%; (h) NaBH_4_, THF/MeOH, 20–30 °C, 91.2%; (i) SOCl_2_/DMF, TCM, 60–70 °C, 97.5%; (j) TMSCN, Pd (OAc)_2_, K_2_CO_3_, 80 °C, 76.2%; (k) NaOH, EtOH; reflux, 88.5%.

The starting material (Figure 5) was the commercially available 2-bromo-3-hydroxy-4-methoxybenzaldehyde. Given that the phenolic hydroxyl group at the 3-position was chemically reactive and prone to participate in subsequent reactions, generating by-products, a methylation reaction was initially conducted. The reaction conditions involved using sodium hydride (2 equivalents) and iodomethane (1.3 equivalents), with DMF selected as the solvents under the temperature of −5 °C to 30 °C [30]. The aldehyde moiety at the C-5 of the intermediate **2-2** was reduced to a hydroxymethyl group. Among the numerous commonly used reductants available in the market, sodium borohydride was opted, which was widely used and required relatively mild reaction conditions for the reaction, and successfully to give Compound **2-3** with a yield of 91.2% [31,32]. The reaction conditions for this step were NaBH_4_ (four equivalents), with the solvent being a mixture of THF and MeOH in a ratio of 1:1, and the temperature ranging from 0 °C to 30 °C. Subsequently, a halogen substitution reaction was performed, where the conversion of benzyl alcohol Compound **2-3** to benzyl chloride Compound **2-4** occurred. [33]. The reaction conditions for this step included thionyl chloride (2 equivalents), with toluene as the solvent, the addition temperature ranging from 0 °C to −5 °C, and the reaction temperature at 25 °C. Next, a cyanation reaction was carried out, by which the substrate underwent a nucleophilic substitution reaction. Next, under the action of palladium acetate, the cyano group was introduced with TMSCN as the cyanide source. [34]. Trimethylsilyl cyanide was used as the cyanating reagent, and Compound **2-5** was successfully prepared. The reaction conditions for this cyanation reaction were TMSCN (2 equivalents), potassium carbonate (3 equivalents) as the acid scavenger, palladium acetate (1 equivalent), and DMF as the solvent, with the reaction being conducted at 80 °C for 12 h. Finally, a hydrolysis reaction was carried out to convert the cyano group into a carboxyl group. The reaction conditions for this hydrolysis step were relatively mild and facile. Specifically, a NaOH solution (4 equivalents) was used with ethanol as the solvent, and the reaction condition was under reflux [32] (Figure 5).

**Figure 6:**
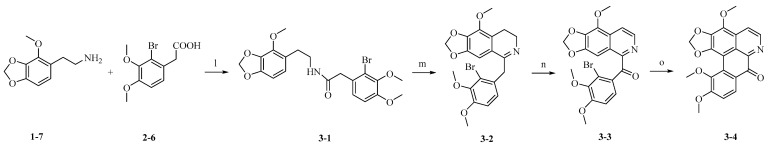
Conditions: (l) EDC/HOBt, DMF, 20–30 °C, 76.8%; (m) POCl_3_, CH_3_CN, reflux, 87.4%; (n) CuBr_2_, DBU, DMSO, 20–30 °C, 78.9%; (o) (1) NaBH_4_, MeOH; (2) 450 W high-pressure mercury lamp (λ = 310 nm); (3) air, 25 °C, 49.0%.

Finally, the condensation of the two key intermediates (**1-7** and **2-6**) was performed (Figure 6) with the reactive groups being amino and carboxyl. Initially, TBTU was employed as the condensing agent and dichloromethane as the solvent for the condensation. However, the reaction outcome was unsatisfactory, and the target compound was not generated. After changing the reaction conditions, the two intermediates were successfully condensed into Compound **3-1** with a yield of 76.8% using EDC and HOBt as condensing agent. The reaction conditions were HOBt (2 equivalents relative to Compound **1-7**), EDC (1.5 equivalents relative to Compound **1-7**), and DMF as the solvent, with the reaction temperature ranging from 0 °C to 30 °C. Subsequently, the obtained amide intermediate **3-1** underwent cyclization [35]. Using phosphorus oxychloride as the cyclizing reagent, Compound 3-2 was successfully synthesized via the Bischler–Napieralski reaction with a yield of 87.4%. The reaction conditions were POCl_3_ (2.0 equivalents relative to Compound 3-1) (2.0 equivalents relative to Compound **3-1**), acetonitrile was selected as the solvent, and the reaction was conducted under reflux at 82 °C [36]. Subsequently, the “one-pot method” was employed to oxidize and aromatize Compound **3-2** to give Compound **3-3**. In the preliminary investigation, we initially adopted chromic acid for oxidation and then utilized Pd/C to catalyze the aromatization reaction according to Cava’s method [36], but failed to reproduce the desired results, and the substrate was scarcely consumed. Therefore, the more convenient “one-pot method” was applied for the oxidation and aromatization [37]. It was noteworthy that DBU and copper bromide need to be added in excess to ensure the completion of the reaction. In summary, the relatively suitable feed ratios and reaction conditions were determined. Specifically, DBU (4 equivalents relative to Compound **3-2**), copper bromide (five equivalents relative to Compound **3-2**), and dried DMSO was selected as the solvent at room temperature. Finally, the photocyclization method was employed for the ring-closing reaction [38]. The bromine ring closure was completed through a three-step reaction including reduction with sodium borohydride, irradiation by a 450-watt mercury lamp, and an oxidizing reaction. Then the target Compound **3-4** was obtained. After comparing the reaction results of mercury lamp irradiations at different wavelengths, the lamp with a wavelength of 310 nm was selected as the optimal reaction condition. Meanwhile, various solvents were compared, such as acetonitrile, methanol, and ethanol, and finally selected more appropriate reaction conditions. The mercury lamp irradiation was finished using a 450-watt lamp with a wavelength of 310 nm and hot methanol as the solvent (Figure 6).

### 2.3. Filiforidine Potently Stimulates Glucose Consumption and Shows No Cytotoxicity in HL-7702 Cells

**Figure 7:**
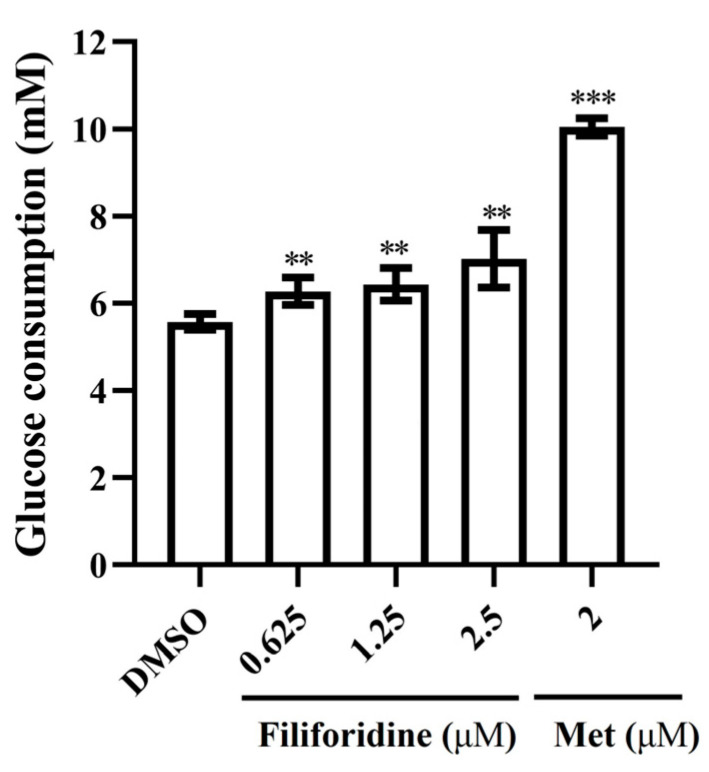
The effects of Filiforidine on glucose in vitro. Glucose consumption values are the means ± SD, n = 5, ** *p* < 0.01 or *** *p* < 0.001 vs. DMSO.

**Figure 8:**
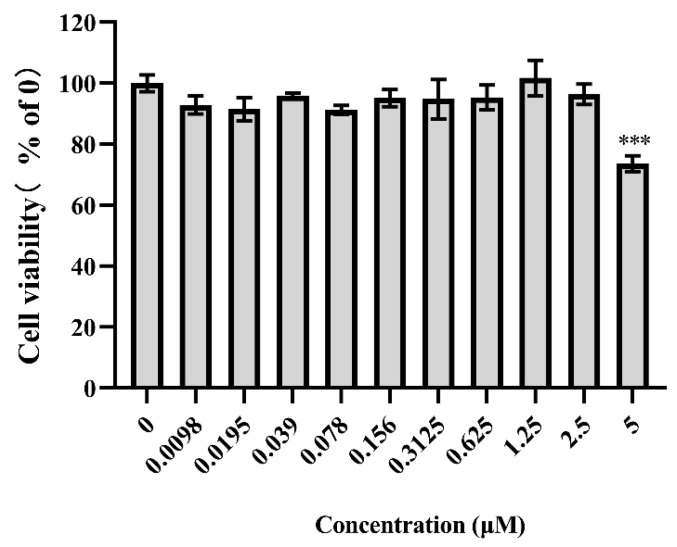
Effects of Filiforidine on cell viability in vitro. HL-7702 cells were incubated with DMSO or Filiforidine at the corresponding concentration for 24 h, and viability was tested by MTT staining. All values are presented as the means ± SD, *** *p* < 0.001 vs. DMSO.

To determine the effects of Filiforidine on cellular glucose consumption, human liver cells of HL-7702 were treated with this compound for 24 h, and Metformin (Met) was used as the positive control. As depicted in Figure 7, Filiforidine caused a significant increase in glucose consumption (*p* < 0.05 vs. DMSO) in HL-7702 at the dosage of 0.625, 1.25 and 2.5 μM. The MTT assay test showed that Filiforidine exhibited significant cytotoxicity at the concentration of 5.0 μM (Figure 8). Compared with the effective dosage of promoting glucose consumption, Filiforidine showed no cytotoxicity against HL-7702 cells.

## 3. Materials and Methods

### 3.1. General

Silica gel (200–300 mesh, Qingdao Marine Chemical Co., Ltd., Qingdao, China) and Sephadex LH-20 (GE Healthcare, Chicago, TL, USA) were used for column chromatography. Reactions were monitored by TLC on silica gel plates (Qingdao Marine Chemical Co., Ltd., GF254, 0.20–0.25 mm) and visualized under UV light (254 nm) using a ZF-20D ultraviolet analyzer (Shanghai Baoshan Gucun Photoelectric Instrument Factory, Shanghai, China). HPLC was performed on a Shimadzu (Tokyo, Japan) LC-16 system equipped with a YMC ODS column (2.5 mm × 250 mm, 5 μm) and an SPD-16 detector, using a methanol–water gradient as the mobile phase. ^1^H and ^13^C NMR spectra were recorded on a JNM-ECZ400S NMR spectrometer (JEOL, Peabody, MA, USA) operating at 400 MHz and 100 MHz, respectively. Chemical shifts (δ) are reported in ppm, and coupling constants (J) are given in Hz. The following abbreviations are used for signal multiplicity: s (singlet), d (doublet), t (triplet), q (quartet), m (multiplet), br (broad), and dd (double doublet). HRMS-ESI data were acquired using an Agilent 1290II mass spectrometer (Agilent Technologies, Santa Clara, CA, USA). HL-7702 cells were obtained from the Cell Bank of the Chinese Academy of Sciences (Shanghai, China). DMEM medium, fetal bovine serum, trypsin, antibiotics, and phosphate-buffered saline were purchased from Gibco-Invitrogen. MTT (thiazolyl blue tetrazolium bromide) was sourced from KeyGEN Biotech (Nanjing, China). Mammalian protein extraction reagent and the BCA protein assay kit were obtained from Thermo Fisher Scientific (Waltham, MA, USA). All other chemicals and reagents were of analytical grade and commercially available.

### 3.2. Plant Material

The whole plant of *C*. *filiformis* was collected from Hainan Province, China, in November 2020. The plant was identified by Professor Niankai Zeng of Hainan Medical University, and a voucher specimen was deposited at the herbarium of the School of Pharmaceutical Science, Hainan Medical University (No. CF202011).

### 3.3. Extraction and Isolation of Filiforidine

The dried *C*. *filiformis* (50 kg) were smashed and extracted under reflux for three times with 95% ethanol as solvent, 2 h each time. The crude extract was vacuum evaporated to recover the solvent until the odor of ethanol disappeared. The pH value of the crude extract was adjusted to 2 by adding 2% HCl and extracted three times with ethyl acetate to remove the liposoluble constituents, followed by adding 1% sodium hydroxide solution, adjusting the pH value to 10, and then successively distributing it in ethyl acetate three times. Total alkaloid (352 g) was obtained after concentration. An aliquot of the total alkaloid (248 g) was subjected to column chromatography eluted with a gradient of CH_2_Cl_2_-CH_3_COCH_3_ (100:0 to 1:1, *v*/*v*) and concentrated under reduced pressure. Finally, six fractions (A-F) were collected. Fraction C was separated on Sephadex LH-20 eluted with CH_3_OH to afford ten subfractions (1–10). Subfra.7 was purified by semi-preparative HPLC eluted with CH_3_OH-H_2_O (65: 35, *v*/*v*, 2.0 mL/min) to give Filiforidine (9.1 mg).

### 3.4. Structural Identification of Filiforidine

UV spectrum was recorded on a JASCO V-650 spectrometer (JASCO Corporation, Tokyo, Japan). IR spectrum was recorded on a Nicolet iS 5 spectrometer (Thermo Fisher Scientific, Madison, WI, USA). One-dimensional (^1^H and ^13^C) NMR spectroscopies and two-dimensional (^1^H-^1^H COSY, HMQC, HMBC) NMR experiments were performed on Bruker (Billerica, MA, USA) AV III 600 spectrometers operating at ^1^H NMR (600 MHz, DMSO-*d*_6_), ^13^C NMR (150 MHz, DMSO-*d*_6_). Chemical shifts were expressed in *δ* (ppm) and coupling constants in Hz. The HRESIMS spectroscopic data were obtained from a Thermo Scientific LTQ Orbitrap XL instrument (Thermo Scientific, Bremen, Germany).

### 3.5. Total Synthesis of Filiforidine

#### 3.5.1. Methoxy-1,3-benzodioxole (**1-2**)

*N*,*N*-dimethylformamide (490 mL), 3-methoxycatechol (**1-1**, 70 g, 0.5 mol, 1.0 eq), diiodomethane (267.8 g, 1.0 mol, 2.0 eq), and potassium carbonate (207 g, 1.50 mol, 3.0 eq) were added in a 1000 mL three-necked flask, sequentially. The mix was heated to 150–160 °C and stirred for 4 h; then, the reaction progress was monitored by TLC. After completion, the reagent was cooled to 20–30 °C, filtered, and concentrated under reduced pressure at 80 °C. The residue in isopropyl ether (300 mL) was dissolved, and the organic phase was washed with distilled water (100 mL × 3), 1.0% sodium hydroxide solution (50 mL, *w*/*v*), and saturated brine (50 mL × 2). The organic phase with anhydrous sodium sulfate (20 g) was dried, filtered, rinsed with isopropyl ether (50 mL), and concentrated under reduced pressure at 45 °C until no droplets condensed. A white solid of 57.1 g was obtained by recrystallization using n-heptane with a yield of 75.0%. ^1^H-NMR (400 MHz, DMSO-*d*_6_): *δ* = 6.75 (d, *J* = 8.0 Hz, 1H), 6.59 (d, *J* = 8.0 Hz, 1H), 6.54 (d, *J* = 8.0 Hz, 1H), 5.92 (s, 2H), 3.77 (s, 3H). ^13^C-NMR (100 MHz, DMSO-*d*_6_): *δ* = 148.8, 144.3, 135.3, 122.6, 108.3, 102.7, 101.4, 56.7. ESI-MS *m*/*z*: 153.6 [M + H]^+^.

#### 3.5.2. 1,3-Benzodioxol-4-ol (**1-3**)

Under nitrogen protection, acetonitrile (700 mL) and 4-methoxy-1,3-benzodioxole (**1-2**, 52 g, 0.34 mol, 1.0 eq) was added to a 2000 mL three-necked flask, and was stirred to dissolve. The reactive reagent was cooled to 0 °C to add sodium iodide (205.3 g, 1.36 mol, 4.0 eq), then add chlorotrimethylsilane (148.6 g, 1.36 mol, 4.0 eq) slowly. After addition, the temperature was maintained at 0–10 °C and stirred for 0.5 h, and then heated to 80–85 °C and stirred for 3 h; then, the reaction progress was monitored by TLC. After completion, the reaction solution was cooled to 20–30 °C, and then the distilled water (100 mL) was slowly added to quench the reaction, followed by isopropyl ether (300 mL) and 10% sodium sulfite solution (100 mL, *w*/*v*). After stirring vigorously for 10 min, phase separation was carried out. The organic phase with saturated sodium chloride solution (50 mL) was washed and then dried over anhydrous sodium sulfate (20 g) for 2 h. After filtering, rinsing with isopropyl ether (50 mL), the filtrate was concentrated under reduced pressure at 45 °C until no droplets condensed. A white solid of 41.4 g was obtained by recrystallization using isopropanol with a yield of 87.6%. ^1^H-NMR (400 MHz, DMSO-*d*_6_): *δ* = 9.58 (s, 1H), 6.60 (d, *J* = 8.0 Hz, 1H), 6.38 (d, *J* = 8.0 Hz, 1H), 6.36 (d, *J* = 8.0 Hz, 1H), 5.87 (s, 2H). ^13^C-NMR (100 MHz, DMSO-*d*_6_): *δ* = 149.0, 141.9, 134.4, 122.3, 111.6, 100.8, 100.8. ESI-MS *m*/*z*: 139.0 [M + H]^+^.

#### 3.5.3. 4-Hydroxy-1,3-benzodioxole-5-carbaldehyde (**1-4**)

Under nitrogen protection, acetonitrile (600 mL), 1,3-benzodioxol-4-ol (**1-3**, 40 g, 0.29 mol, 1.0 eq), anhydrous magnesium chloride (41.4 g, 0.43 mol, 1.5 eq), and triethylamine (49.7 g, 0.49 mol, 1.7 eq) were added sequentially to a 1000 mL three-necked flask. The mixture was heated to 65 °C and stirred for 0.5 h, then paraformaldehyde (34.8 g, 1.16 mol, 4.0 eq) was added. The temperature was slowly raised to 82 °C and stirred for 4 h. After completion, the reaction solution was concentrated under reduced pressure and cooled to 20–30 °C; then, distilled water (100 mL) was added, and the pH was adjusted to **2-3** with 10% hydrochloric acid (10 mL, *w*/*v*). Then, the solvent was extracted with ethyl acetate (100 mL × 3), after combining the organic phases, and washed with distilled water (100 mL × 3) and saturated sodium chloride solution (100 mL × 2). The organic phase was dried over anhydrous sodium sulfate (50 g) for 2 h. The solution was filtered, rinsed with ethyl acetate (50 mL), concentrated under reduced pressure at 45 °C, and purified by recrystallization using n-hexane to obtain 37.5 g of white solid, yielding 78.0%. ^1^H-NMR (400 MHz, DMSO-*d*_6_): *δ* = 10.80 (s, 1H), 9.93 (s, 1H), 7.72 (d, *J* = 8.0 Hz, 1H), 6.66 (d, *J* = 8.0 Hz, 1H), 6.11 (s, 2H). ^13^C-NMR (100 MHz, DMSO-*d*_6_): *δ* = 102.8, 154.7, 144.7, 137.8, 128.0, 119.9, 103.0, 102.3. ESI-MS *m*/*z*: 165.0 [M − H]^−^.

#### 3.5.4. 4-Methoxy-1,3-benzodioxole-5-carbaldehyde (**1-5**)

*N*,*N*-dimethylformamide (200 mL) and 4-hydroxy-1,3-benzodioxole-5-carbaldehyde (**1**-**4**, 35 g, 0.21 mol, 1.0 eq) were added into a 1000 mL single-necked flask, and the solvent was stirred to dissolve. The reaction solution was cooled to 0 °C, and then sodium hydride (12.7 g, 0.32 mol, 1.5 eq) was added. After stirring for 0.5 h and warming to 20–30 °C, iodomethane (38.9 g, 0.27 mol, 1.3 eq) was slowly added. The reaction was conducted by shielding from light and react for 4 h, and then progress was monitored by TLC. After completion, the solution was cooled to 0 °C, quenched with distilled water (50 mL), and extracted with ethyl acetate (100 mL × 3). The organic phases were washed with distilled water (100 mL) and saturated sodium chloride solution (50 mL × 2), and then dried over anhydrous sodium sulfate (20 g) for 4 h. The reaction solution was filtered, rinsed with ethyl acetate (20 mL), concentrated under reduced pressure at 45 °C until no droplets condensed, and purified by recrystallization with isopropyl ether to obtain 31.1 g of white solid, yielding 82.0%. ^1^H-NMR (400 MHz, DMSO-*d*_6_): *δ* = 10.06 (s, 1H), 7.31 (d, *J* = 8.0 Hz, 1H), 6.75 (d, *J* = 8.0 Hz, 1H), 6.11 (s, *J* = 8.0 Hz, 2H), 4.02 (s, 3H). ^13^C-NMR (100 MHz, DMSO-*d*_6_): *δ* = 188.0, 155.1, 146.0, 136.8, 124.3, 123.0, 104.1, 102.9, 60.8. ESI-MS *m*/*z*: 181.0 [M + H]^+^.

#### 3.5.5. (*E*)-4-Methoxy-5-(2-nitrovinyl)-1,3-benzodioxole (**1-6**)

4-methoxy-1,3-benzodioxole-5-carbaldehyde (**1-5**, 30 g, 0.167 mol, 1.0 eq), acetic acid (200 mL), ammonium acetate (19.2 g, 0.25 mol, 1.5 eq), and nitromethane (40.7 g, 0.67 mol, 4.0 eq) were added into a 1000 mL three-necked flask. The mixture was heated to 118 °C and refluxed for 5 h, and then progress was monitored by TLC. After completion, the solution was cooled to 60–70 °C and the solvent was removed under reduced pressure. After cooling to 20–30 °C, adding anhydrous ethanol (150 mL), stirring for crystallization (3–4 h), washing with purified water until neutral, and then drying at 40–50 °C, a yellow solid of 35.1 g was obtained with a yield of 87.0%. ^1^H-NMR (400 MHz, DMSO-*d*_6_): *δ* = 8.07 (d, *J* = 16.0 Hz, 1H), 7.98 (d, *J* = 16.0 Hz, 1H), 7.40 (d, *J* = 8.0 Hz, 1H), 6.74 (d, *J* = 8.0 Hz, 1H), 6.08 (s, 2H), 4.04 (s, 3H). ^13^C-NMR (100 MHz, DMSO-*d*_6_): *δ* = 153.5, 143.6, 137.0, 136.9, 135.6, 127.4, 116.4, 104.3, 102.7, 60.5. ESI-MS *m*/*z*: 224.6 [M + H]^+^.

#### 3.5.6. β-(2-Methoxy-3,4-methylenedioxyphenyl) ethylamine (**1-7**)

Dried tetrahydrofuran (170 mL) and lithium aluminum hydride (22.2 g, 0.6 mol, 4.0 eq) were added into a 1000 mL three-necked flask. The mixture was cooled to 0 °C and a solution of (*E*)-4-methoxy-5-(2-nitrovinyl)-1,3-benzodioxole (**1-6**, 33.5 g, 0.15 mol, 1.0 eq, dissolved in 170 mL THF) was slowly added over 4 h while maintaining the temperature at 0–5 °C. After addition, the reaction solution was stirred at 0 °C for 0.5 h, and then warmed to 30–40 °C and stirred for 3 h, monitoring by TLC. After completion, the solvent was cooled to 0 °C and slowly quenched with purified water (22 mL), followed by 15% NaOH (22 mL). The solution was filtered with celite and concentrated under reduced pressure at 45 °C. Distilled water (100 mL) was added, and the solution was extracted with isopropyl ether (150 mL × 3). The organic phase was stirred with 10% citric acid (370 mL, *w*/*v*) for 10 min. After phase separation, the aqueous phase was adjusted to pH 7.0–7.5 with saturated sodium bicarbonate, back-extract with isopropyl ether (100 mL × 3), then the aqueous phase was adjusted to pH 10–11 with 10% NaOH (80 mL, *w*/*v*). The aqueous phase was extracted with ethyl acetate (100 mL × 3), washed with distilled water (50 mL × 2) and saturated sodium chloride solution (50 mL × 2), and dried over anhydrous sodium sulfate (20 g) for 2 h. The solvent was filtered, rinsed with ethyl acetate (20 mL), and concentrated under reduced pressure at 45 °C to obtain 21.5 g of yellow oil, yielding 73.3%. ^1^H-NMR (400 MHz, DMSO-*d*_6_): *δ* = 6.57 (d, *J* = 8.0 Hz, 1H), 6.48 (d, *J* = 8.0 Hz, 1H), 5.90 (s, 2H), 3.86 (s, 3H), 2.60–2.64 (m, 2H), 2.50–2.53 (m, 2H). ^13^C-NMR (100 MHz, DMSO-*d*_6_): *δ* = 147.9, 141.9, 137.0, 125.7, 123.0, 103.0, 101.3, 59.9, 43.2, 34.4. ESI-MS *m*/*z*: 196.1 [M + H]^+^.

#### 3.5.7. 2-Bromo-3,4-dimethoxybenzaldehyde (**2-2**)

A 1000 mL three-necked flask was charged with 2-bromo-3-hydroxy-4-methoxybenzaldehyde (**2-1**, 46.2 g, 0.2 mol, 1.0 eq) and *N*,*N*-dimethylformamide (200 mL). The mixture was cooled to 0 °C, and sodium hydride (60%, 12 g, 0.3 mol, 1.5 eq) was added in portions, followed by stirring for 30 min. Iodomethane (37 g, 0.26 mol, 1.3 eq) was then added dropwise, and the reaction was allowed to proceed at 0–10 °C in the dark for 4 h. TLC monitoring confirmed the complete consumption of the starting material. The reaction mixture was cooled to 0 °C, and distilled water (200 mL) was slowly added to quench the reaction. The mixture was extracted with ethyl acetate (100 mL × 3), and the combined organic phases were washed with distilled water (50 mL) and saturated sodium chloride solution (50 mL), and then dried over anhydrous sodium sulfate (20 g). After filtration and washing with ethyl acetate (20 mL), the solution was concentrated under reduced pressure at 45 °C until no liquid droplets condensed in the condenser. Recrystallization was conducted using n-hexane to obtain 42.1 g of an off-white solid, with a yield of 86.0%. ^1^H-NMR (400 MHz, DMSO-*d*_6_): *δ* = 10.05 (s, 1H), 7.62 (d, *J* = 8.0 Hz, 1H), 7.22 (d, *J* = 8.0 Hz, 1H), 3.90 (s, 3H), 3.73 (s, 3H). ^13^C-NMR (100 MHz, DMSO-*d*_6_): *δ* = 190.9, 159.0, 146.3, 127.4, 127.2, 122.0, 112.7, 60.7, 57.1. ESI-MS *m*/*z*: 246.0 [M + H]^+^.

#### 3.5.8. 2-Bromo-3,4-dimethoxybenzyl alcohol (**2-3**)

A 1000 mL three-necked flask was charged sequentially with 2-bromo-3,4-dimethoxybenzaldehyde (**2-2**, 39.1 g, 0.16 mol, 1.0 eq), tetrahydrofuran (80 mL), and methanol (80 mL). The mixture was cooled to 0 °C, and sodium borohydride (18.1 g, 0.48 mol, 3.0 eq) was added in portions with stirring. The temperature was then raised to 20–30 °C, and the mixture was stirred for 4 h, with progress monitored by TLC. Upon completion, distilled water (80 mL) was added to quench the reaction. The solvent was removed under reduced pressure at 40 °C, and the residue was extracted with isopropyl ether (200 mL × 3). The combined organic phases were washed with distilled water (100 mL × 3) and saturated sodium chloride solution (100 mL × 2), and then dried over anhydrous sodium sulfate (20 g). After filtration and washing with isopropyl ether (50 mL), the solution was concentrated under reduced pressure at 45 °C until no liquid droplets condensed. The residue was recrystallized from isopropanol to yield 36 g of a white solid, with a yield of 91.2%. ^1^H-NMR (400 MHz, DMSO-*d*_6_): *δ* = 7.18 (d, *J* = 8.0 Hz, 1H), 7.04 (d, *J* = 8.0 Hz, 1H), 5.25 (m, 1H), 4.40 (d, *J* = 4.0 Hz, 2H), 3.77 (s, 3H), 3.67 (s, 3H). ^13^C-NMR (100 MHz, DMSO-*d*_6_): *δ* = 152.6, 145.8, 134.2, 123.7, 117.0, 112.3, 63.0, 60.4, 56.6. ESI-MS *m*/*z*: 247.0 [M + H]^+^.

#### 3.5.9. 2-Bromo-1-(chloromethyl)-3,4-dimethoxybenzene (**2-4**)

Chloroform (150 mL), 2-bromo-3,4-dimethoxybenzyl alcohol (**2-3**, 35 g, 0.14 mol, 1.0 eq), and *N*,*N*-dimethylformamide (3.5 g) were added into a 500 mL three-necked flask. The mixture was stirred to dissolve and then cooled to 0 °C in an ice bath. Thionyl chloride (25 g, 0.21 mol, 1.5 eq) was slowly added dropwise, and the temperature was raised to 60–70 °C. The reaction was stirred for 2 h, with progress monitored by TLC. After completion, the mixture was concentrated under reduced pressure at 40 °C. The residue was dissolved in chloroform (150 mL), washed with distilled water (30 mL × 2), saturated sodium bicarbonate solution (30 mL), and saturated sodium chloride solution (20 mL), and then dried over anhydrous sodium sulfate (10 g). After filtration, the solution was concentrated under reduced pressure at 45 °C to yield a yellow oil (36.7 g), with a calculated yield of 97.5%. ^1^H-NMR (400 MHz, DMSO-*d*_6_): *δ* = 7.31 (d, *J* = 8.0 Hz, 1H), 7.04 (d, *J* = 8.0 Hz, 1H), 4.74 (s, 2H), 3.80 (s, 3H), 3.60 (s, 3H). ^13^C-NMR (100 MHz, DMSO-*d*_6_): *δ* = 154.2, 146.6, 129.7, 127.5, 120.0, 112.5, 60.5, 56.6, 47.4. ESI-MS *m*/*z*: 264.0 [M + H]^+^.

#### 3.5.10. 2-(2-Bromo-3,4-dimethoxyphenyl) acetonitrile (**2-5**)

*N*,*N*-dimethylformamide (200 mL) and 2-bromo-1-(chloromethyl)-3,4-dimethoxybenzene (**2-4**, 35.3 g, 0.133 mol, 1.0 eq) were added into a 500 mL three-necked flask. The mixture was stirred to dissolve, followed by the addition of potassium carbonate (55 g, 0.4 mol, 3.0 eq), trimethylsilyl cyanide (TMSCN, 26.4 g, 0.266 mol, 2.0 eq), and palladium acetate (1.3 g). The temperature was raised to 80 °C and the reaction was stirred for 12 h, and progress was monitored by TLC. After completion, the mixture was cooled to 20–30 °C, filtered, and the filtrate was poured into ice water (600 mL). The mixture was stirred thoroughly and then filtered. The filter cake was washed with distilled water (400 mL) and recrystallized from a hexane-isopropyl ether mixture to yield 25.9 g of a white solid, with a calculated yield of 76.2%. ^1^H-NMR (400 MHz, DMSO-*d*_6_): *δ* = 7.23 (d, *J* = 8.0 Hz, 1H), 7.08 (d, *J* = 8.0 Hz, 1H), 3.95 (s, 2H), 3.80 (s, 3H), 3.70 (s, 3H). ^13^C-NMR (100 MHz, DMSO-*d*_6_): *δ* = 153.5, 146.7, 126.0, 123.8, 119.3, 118.7, 112.9, 60.5, 56.7, 24.1. ESI-MS *m*/*z*: 256.0 [M + H]^+^.

#### 3.5.11. 2-(2-Bromo-3,4-dimethoxyphenyl) acetic acid (**2-6**)

Absolute ethanol (150 mL) was transferred into a 250 mL three-necked flask, followed by adding 2-(2-bromo-3,4-dimethoxyphenyl) acetonitrile (**2-5**, 25.6 g, 0.1 mol, 1.0 eq) to the flask. The mixture was stirred to dissolve, and then an aqueous NaOH solution (12 g in 50 mL, 3.0 eq) was added. The reaction was heated under reflux for 24 h, with progress monitored by TLC. After completion, the mixture was cooled to 40 °C. Ethanol was removed under reduced pressure, and then distilled water (100 mL) was added. The mixture was extracted with ethyl acetate (30 mL), and the aqueous phase was acidified to pH 2–3 by dropwise addition of 10% hydrochloric acid (110 mL). A solid precipitated, which was filtered and washed with distilled water until neutral. The filter cake was dried under vacuum at 40–50 °C to yield a white of 24.3 g, with a calculated yield of 88.5%. ^1^H-NMR (400 MHz, CDCl_3_): *δ* = 6.70 (d, *J* = 8.0 Hz, 1H), 6.83 (d, *J* = 8.0 Hz, 1H), 3.85 (s, 3H), 3.84 (s, 3H), 3.78 (s, 2H). ^13^C-NMR (100 MHz, CDCl_3_): *δ* = 176.7, 153.0, 146.8, 126.6, 126.4, 121.0, 111.3, 60.5, 56.2, 41.0. ESI-MS *m*/*z*: 275.0 [M + H]^+^.

#### 3.5.12. *N*-[2-(4-Methoxy-1,3-methylenedioxyphenyl-5-yl)ethyl]-3,4-dimethoxy-2-bromophenethylamide (**3-1**)

Dried *N*,*N*-dimethylformamide (200 mL), 2-(2-bromo-3,4-dimethoxyphenyl) acetic acid (**2-6**, 27.4 g, 0.1 mol, 1.0 eq), and β-(2-methoxy-3,4-methylenedioxyphenyl) ethylamine (1-7, 19.5 g, 0.1 mol, 1.0 eq) were added into a 1000 mL three-necked flask. The mixture was stirred to dissolve, followed by the addition of 1-hydroxybenzotriazole (HOBt, 27 g, 0.2 mol, 2.0 eq). The temperature was lowered to 0 °C, and 1-ethyl-(3-dimethylaminopropyl) carbodiimide (EDC, 38.3 g, 0.2 mol, 2.0 eq) was added. The reaction was stirred at 0 °C for 20 min, and then warmed to 20–30 °C and stirred for 3 h. TLC was used to verify the complete consumption of the starting material. The reaction was quenched with 200 mL of ammonium chloride solution, and the pH was adjusted to 2–3 with 10% hydrochloric acid (100 mL, *w*/*v*). The mixture was extracted with ethyl acetate (150 mL × 3), and the combined organic phases were washed with distilled water (100 mL × 4) and 2.0% sodium bicarbonate (100 mL), then dried over anhydrous sodium sulfate (50 g) for 4 h. After filtration and washing with ethyl acetate (50 mL), the solvent was concentrated under reduced pressure at 40 °C to about half its volume. Crystallization was induced at 0 °C for 2–3 h, followed by filtration and drying under vacuum at 40 °C to afford a white solid of 34.7 g (yield: 76.8%). ^1^H-NMR (400 MHz, CDCl_3_): *δ* = 6.97 (d, *J* = 8.0 Hz, 1H), 6.80 (d, *J* = 8.0 Hz, 1H), 6.41 (d, *J* = 8.0 Hz, 1H), 6.35 (d, *J* = 8.0 Hz, 1H), 5.87 (s, 2H), 3.88 (s, 3H), 3.85 (s, 3H), 3.81 (s, 3H), 3.58 (s, 2H), 3.34–3.37 (m, 2H), 2.61–2.66 (m, 2H). ^13^C-NMR (100 MHz, CDCl_3_): *δ* = 170.1, 152.9, 148.2, 146.9, 141.7, 136.5, 127.8, 126.6, 124.0, 122.9, 120.9, 115.5, 102.6, 100.9, 60.5, 59.7, 56.2, 43.7, 40.3, 29.9. ESI-MS *m*/*z*: 474.1 [M + H]^+^.

#### 3.5.13. 5-[(2-Bromo-3,4-dimethoxyphenyl) methyl]-9-methoxy-7,8-dihydro-[1,3]dioxolo [4,5-g]isoquinoline (**3-2**)

Dried acetonitrile (150 mL) and *N*-[2-(4-methoxy-1,3-methylenedioxyphenyl-5-yl) ethyl]-3,4-dimethoxy-2-bromophenethylamide (**3-1**, 33.1 g, 73 mmol, 1.0 eq) were added into a 250 mL three-necked flask. The mixture was stirred to dissolve, followed by the addition of phosphorus oxychloride (22.4 g, 146 mmol, 2.0 eq). The reaction was refluxed for 3 h, with progress monitored by TLC. After completion, the mixture was cooled to 40 °C and concentrated under reduced pressure until no more distillate was observed. The temperature was adjusted to 20–30 °C, and saturated sodium bicarbonate solution (100 mL) was added dropwise to neutralize the mixture. The product was extracted with ethyl acetate (100 mL × 3), and the combined organic phases were washed with distilled water (100 mL) and saturated sodium chloride solution (100 mL × 2), then dried over anhydrous sodium sulfate (30 g) for 2 h. After filtration and washing with ethyl acetate (50 mL), the solution was concentrated under reduced pressure at 45 °C to afford a yellow solid of 27.8 g (yield of 87.4%). ^1^H-NMR (400 MHz, CDCl_3_): *δ* = 6.88 (d, *J* = 8.0 Hz, 1H), 6.75 (d, *J* = 8.0 Hz, 1H), 6.68 (s, 1H), 5.92 (s, 2H), 4.12 (s, 2H), 3.94 (s, 3H), 3.90 (s, 3H), 3.81 (s, 3H), 3.64–3.70 (m, 2H), 2.65–2.69 (m, 2H). ^13^C-NMR (100 MHz, CDCl_3_): *δ* = 165.0, 152.2, 147.6, 146.6, 139.8, 138.2, 130.5, 124.9, 123.3, 120.5, 111.4, 101.3, 100.9, 60.5, 59.8, 56.1, 46.9, 42.3, 19.4. ESI-MS *m*/*z*: 434.1 [M + H]^+^.

#### 3.5.14. (2-Bromo-3,4-dimethoxyphenyl) (9-methoxy-[1,3]dioxolo[4,5-g] isoquinolin-5-yl) methanone (**3**-**3**)

Dimethyl sulfoxide (100 mL), 5-[(2-bromo-3,4-dimethoxyphenyl)methyl]-9-methoxy-[1,3]dioxolo[4,5-g]isoq-unoline (**3-2**, 21.6 g, 50 mmol, 1.0 eq), 1,8-diazabicyclo[5.4.0]undec-7-ene (DBU, 30.4 g, 0.2 mol, 4.0 eq), and copper (II) bromide (55.8 g, 0.25 mol, 5.0 eq) were added into a 250 mL three-necked flask. The mixture was stirred at 20–30 °C for 4 h, with progress monitored by TLC. After completion, the reaction was quenched by dropwise addition of sodium bicarbonate solution (100 mL). The product was extracted with ethyl acetate (100 mL × 3), and the combined organic phases were washed with distilled water (100 mL × 3) and saturated sodium chloride solution (100 mL × 2), then dried over anhydrous sodium sulfate (20 g) overnight. After filtration and concentration under reduced pressure at 45 °C, a yellow solid of 17.6 g was obtained (yield: 78.9%). ^1^H-NMR (400 MHz, CDCl_3_): *δ* = 8.35 (d, *J* = 4.0 Hz, 1H), 7.99 (d, *J* = 8.0 Hz, 1H), 7.73 (s, 1H), 7.40 (d, *J* = 12.0 Hz, 1H), 6.95 (d, *J* = 8.0 Hz, 1H), 6.09 (s, 2H), 4.20 (s, 3H), 3.92 (s, 3H), 3.83 (s, 3H). ^13^C-NMR (100 MHz, CDCl_3_): *δ* = 169.1, 179.2, 156.1, 152.6, 151.2, 146.5, 140.2, 136.7, 135.3, 134.3, 130.4, 127.6, 124.5, 117.9, 110.9, 101.8, 97.1, 60.0, 60.4, 56.2. ESI-MS *m*/*z*: 446.0 [M + H]^+^.

#### 3.5.15. 3,10,11-Trimethoxy-1,2-methylenedioxy-7-oxoaporphine (Filiforidine, **3**-**4**)

Sodium borohydride (1.9 g, 46.0 mmol, 1.5 eq) was added in portions to a hot methanol solution of (2-bromo-3,4-dimethoxyphenyl) (9-methoxy-[1,3]dioxolo[4,5-g] isoquinolin-5-yl) methanone (**3**-**3**, 15 g, 33.7 mmol, 1.0 eq) under stirring. After complete addition, the reaction was stirred for 0.5 h, and TLC confirmed full consumption of **3**-**3**. The mixture was cooled to 20–30 °C and irradiated with a 450 W high-pressure mercury lamp (λ = 310 nm) for 3 h, followed by air oxidation for 1 h. TLC (CH_2_Cl_2_: MeOH = 10:1, Rf = 0.65) was applied to indicate reaction completion. The mixture was concentrated under reduced pressure, and the residue was dissolved in dichloromethane (100 mL), washed with distilled water (30 mL × 3), and dried over anhydrous sodium sulfate (10 g). After filtration and concentration at 40 °C, the crude product was purified by silica gel column chromatography (CH_2_Cl_2_: MeOH = 20:1) to afford 6 g of a yellow solid (yield: 49%). ^1^H-NMR (400 MHz, DMSO-*d*_6_): *δ* = 8.80 (d, *J* = 7.2 Hz, 1H), 8.15 (d, *J* = 7.2 Hz, 1H), 8.12 (d, *J* = 8.8 Hz, 1H), 7.39 (d, *J* = 8.8 Hz, 1H), 6.40 (s, 2H), 3.84 (s, 3H), 4.00 (s, 3H), 4.25 (s, 3H). ^13^C-NMR (100 MHz, DMSO-*d*_6_): *δ* = 180.6, 158.6, 150.0, 146.0, 144.4, 144.2, 138.6, 135.8, 130.5, 126.5, 126.1, 125.3, 124.5, 118.3, 113.2, 102.7, 99.4, 61.3, 57.0. ESI-MS *m*/*z*: 366.1 [M + H]^+^.

### 3.6. Cytotoxicity Assay

Cell viability was evaluated by the MTT assay. In brief, HL-7702 cells were seeded into 96-well plates, cultured as above. Then cells were treated with Filiforidine or metformin (Met) at different concentration for 24h. After treatments, the media were removed and the cells were incubated with 50 μL MTT solution for 4 h. The supernatant was aspirated, and 150 μL of DMSO was added per well and dissolved with a plate shaker for 5 min. The level of MTT formazan was determined by measuring its absorbance at 490 nm with a microplate reader.

### 3.7. Glucose Consumption Assay

After treatment, culture supernatants were harvested, the glucose levels were assayed by the Randox glucose assay kit based on the glucose oxidase method (Beijing Strong Biotechnologies, Inc., Beijing, China), and glucose consumption was calculated by the initial glucose level minus the remaining glucose level of medium.

### 3.8. Statistical Analysis

For the above experiments, our values are displayed as the mean ± SD with three independent repetitions. GraphPad Prism 8.0 software was used to process and analyze data. The differences among the studied groups were assessed by *t* test or one-way ANOVA, and *p* < 0.05 was considered statistically significant.

## 4. Conclusions

The treatment of type 2 diabetes (T2DM) requires lifelong medication. Among the many medications available to treat diabetes, sulfonylureas are the first choice for the early treatment of patients with T2DM and function by stimulating pancreatic β-cells to secrete more insulin, along with insulin sensitizers, carbohydrate-inhibiting drugs, insulin, enzyme target modulators and herbal remedies [39,40]. Although there are a wide range of clinical drug treatments for T2MD, different drugs have their own advantages and disadvantages. For example, there are side effects such as short duration of effect and low potency leading to uncontrolled blood glucose, and long-term use can manifest as hypoglycemia, hyperthermia, gastrointestinal discomfort and swelling [41]. To date, there is still no cure for type 2 diabetes, with interventions focused on glycemic control and the management of associated complications. There is therefore still an urgent need for new effective hypoglycemic agents to gain more benefits and treatment options. Diabetes is a chronic lifelong disease. In clinical practice, it is mostly treated with Western drugs, which are fast acting and effective. However, they are susceptible to drug resistance in diabetic patients, thus causing different toxicities and side effects, such as hypoglycemia and hyperalgesia, which are detrimental to long-term use in diabetic patients. Therefore, screening and research in natural products to develop hypoglycemic drugs with low toxicities and side effects have become a hot research topic [42,43].

Ethnic medicine, belonging to TCM, is a kind of medicine developed by the ethnic minorities in China during their long struggle with diseases. It has a long history of preventing and treating diabetes, and is characterized by precise efficacy, moderate efficacy and low toxic side effects. Some studies have found that ethnic medicines are one of the most important sources of anti-diabetic drugs, as they are rich in varieties, resources and natural products with significant activity [44]. *C*. *filiformis* is a well-known ethnic medicine in the Li region of Hainan Province, China, used folklorically as a treatment for diabetes and other diseases [45]. Researchers used the methanolic extract of *C*. *filiformis* to treat mice with alloxan-induced diabetes and showed a 46.8% reduction in blood glucose [14]. The *C*. *filiformis* extract can modulate the transport of glibenclamide through the inhibition of P-glycoprotein to exert an anti-diabetic effect [46]. Studies have shown that the main constituent of *C*. *filiformis* extract is the aporphine alkaloids. The antidiabetic effects of some aporphine alkaloids have been studied. The results of the studies show good promise for application [47,48]. However, the antidiabetic effects of the aporphine alkaloids in *C*. *filiformis* have not been fully discovered. Therefore, the aporphine alkaloids rich in *C*. *filiformis* attracted our research interest. In this study, Filiforidine was obtained through a series of chemical chromatographic methods. Furthermore, a complex post-treatment method was developed, which reduced the column chromatography separation steps. Specifically, 2-(4-methoxybenzo[d][1,3]dioxol-5-yl) ethan-1-amine is salted with dilute hydrochloric acid.

The basic feature of diabetes is an increase in blood glucose and the principle of treatment is to control blood glucose levels [49]. We tested Filiforidine in HL-7702 cells and found that Filiforidine at 0.625, 1.25 and 2.5 μM increased the relative glucose consumption in a dose-dependent and non-cytotoxic manner. This suggests that Filiforidine may increase the ability of hepatocytes to use glucose to improve diabetes. We highlight that this study not only isolates the Filiforidine but also establishes the total synthesis for the first time.

## Figures and Tables

**Table 1:** NMR data of Filiforidine (*δ* in ppm, *J* values in Hz).

No.	*δ*_c_	*δ*_H_	No.	*δ*_c_	*δ*_H_
1	150.4	-	7a	126.5	-
1a	99.8	-	8	125.3	8.07, d, (8.8)
1b	123.2	-	9	113.2	7.34, d, (8.8)
2	138.6	-	10	158.3	-
3	135.8	-	11	146.0	-
3a	130.5		11a	126.2	-
4	144.5	8.76, d, (5.2)	3-OMe	60.8	4.21, s
5	118.6	8.10, d, (5.2)	10-OMe	56.8	3.96, s
6a	144.2	-	11-OMe	61.0	3.80, s
7	180.9	-	O-CH_2_-O	103.1	6.36, s

Measured at 600 MHz for ^1^H NMR and 150 MHz for ^13^C NMR in DMSO-*d*_6_.

## Data Availability

Data is contained within the article or Supplementary Material.

## Supplementary Materials

The following supporting information can be downloaded at: https://www.mdpi.com/article/10.3390/molecules30244763/s1. The NMR (^1^H and ^13^C), UV, IR, and MS data for Filiforidine, along with the original ^1^H and ^13^C NMR spectra for each synthetic step.
