# Supplementary material for: Isolation, Total Synthesis and Anti-Diabetic Activity of Filiforidine from Cassytha filiformis
Source: Molecules. 2025 Dec 12;30(24):4763. doi: 10.3390/molecules30244763 (PMC12736250; doi:10.3390/molecules30244763)
Supplement: Supplementary file 1 [file molecules-30-04763-s001.zip › molecules-4006965-supplementary.pdf]

molecules

Supporting Information for

Isolation, Total Synthesis and Anti-Diabetic Activity of Filiforidine from *Cassytha filiformis*

Caiyun Zhang [1,2,†], Hong Zhu [2,†], Fang Zhang [2], Yuexia Jiang [2], Zibao Huang [2], Dong Lin [2], Niangen Chen [2,*] and Xiaopo Zhang [2,*] and Yanhui Fu [1,*]

[1] College of Chemistry and Chemical Engineering, Hainan Normal University, Haikou 571158, China; hy0308018@muhn.edu.cn
[2] School of Pharmaceutical Science, Hainan Medical University, Haikou 571199, China; 18789928157@163.com (H.Z.); hy0207102@muhn.edu.cn (F.Z.); hy0207025@muhn.edu.cn (Y.J.); huangzibao0107@163.com (Z.H.); donglin793@126.com (D.L.)
* Correspondence: hy0207011@muhn.com (N.C.); hy0207077@muhn.edu.cn (X.Z.); fuyanhui80@163.com (Y.F.)
† These authors contributed equally to this work.

Table of Contents

- ^{1}H NMR and ^{13}C NMR spectrum of Filiforidine…… S2
- HMQC and HMBC spectrum of Filiforidine…… S3
- ^{1}H-^{1}H COSY spectrum of Filiforidine…… S4
- HPLC of Filiforidine…… S4
- The UV spectrum of Filiforidine……S4
- The IR and mass spectrum of Filiforidine……S5
- ^{1}H NMR and ^{13}C NMR spectra of **1-2**…… S6
- ^{1}H NMR and ^{13}C NMR spectra of **1-3**…… S7
- ^{1}H NMR and ^{13}C NMR spectra of **1-4**…… S8
- ^{1}H NMR and ^{13}C NMR spectra of **1-5**…… S9
- ^{1}H NMR and ^{13}C NMR spectra of **1-6**…… S10
- ^{1}H NMR and ^{13}C NMR spectra of **1-7**…… S11
- ^{1}H NMR and ^{13}C NMR spectra of **2-2**…… S12
- ^{1}H NMR and ^{13}C NMR spectra of **2-3**…… S13
- ^{1}H NMR and ^{13}C NMR spectra of **2-4**…… S14
- ^{1}H NMR and ^{13}C NMR spectra of **2-5**…… S15
- ^{1}H NMR and ^{13}C NMR spectra of **2-6**…… S16
- ^{1}H NMR and ^{13}C NMR spectra of **3-1**…… S17
- ^{1}H NMR and ^{13}C NMR spectra of **3-2**…… S18
- ^{1}H NMR and ^{13}C NMR spectra of **3-3**…… S19
- ^{1}H NMR and ^{13}C NMR spectra of **3-4**…… S20

^{1}H-NMR spectrum of Filiforidine

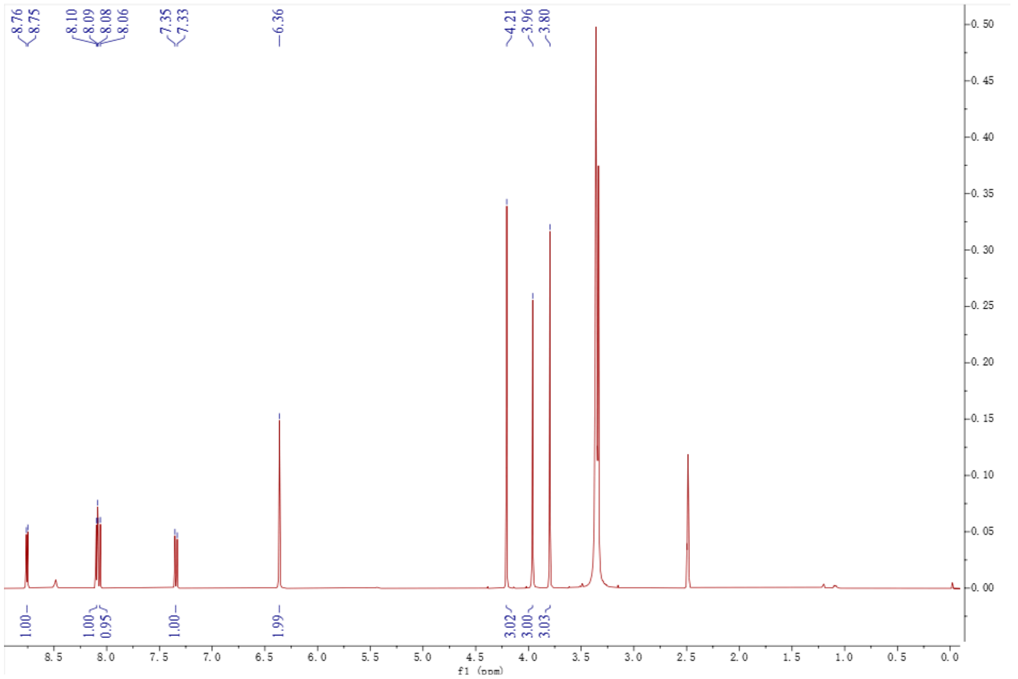

^{13}C-NMR spectrum of Filiforidine

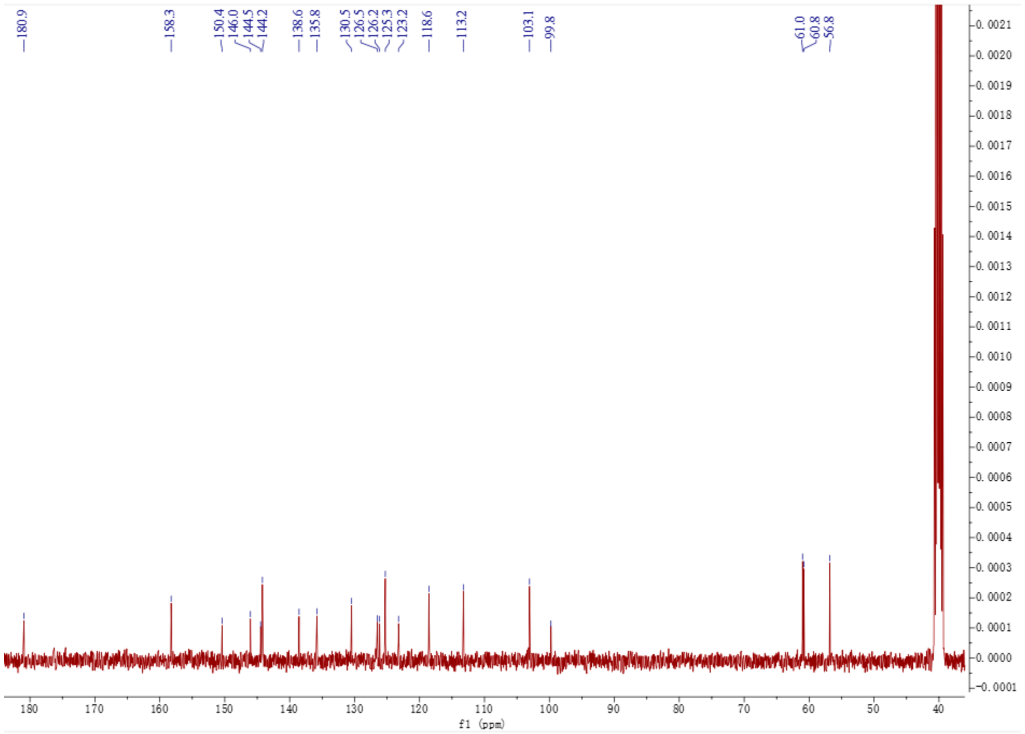

HMQC spectrum of Filiforidine

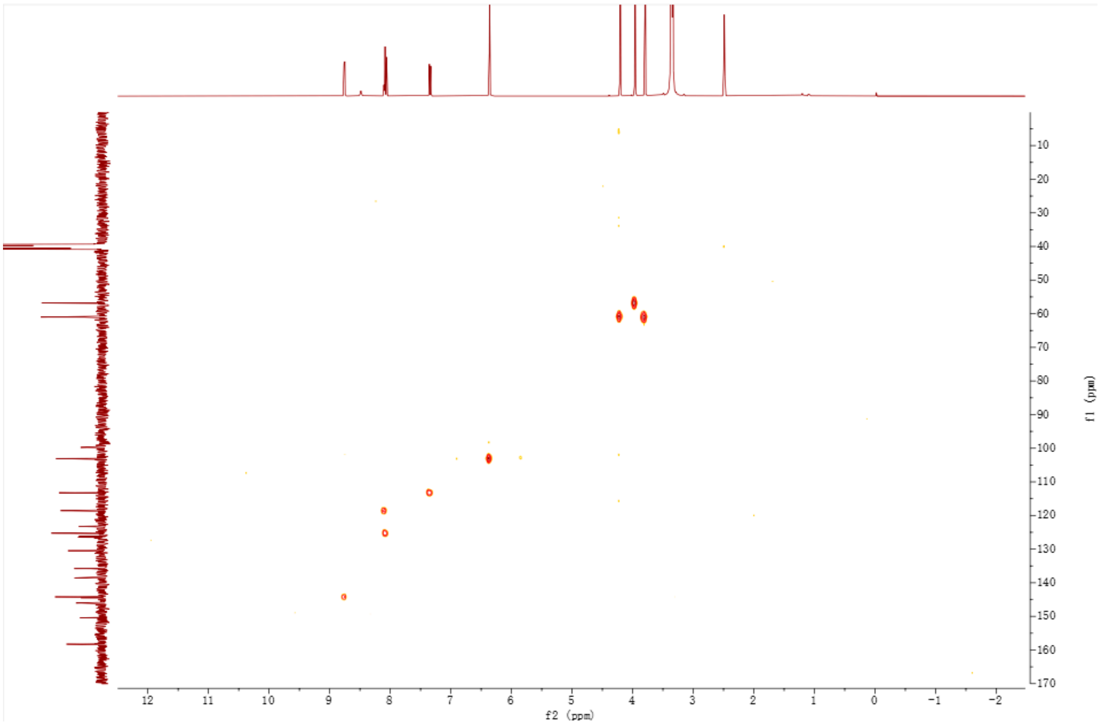

HMBC spectrum of Filiforidine

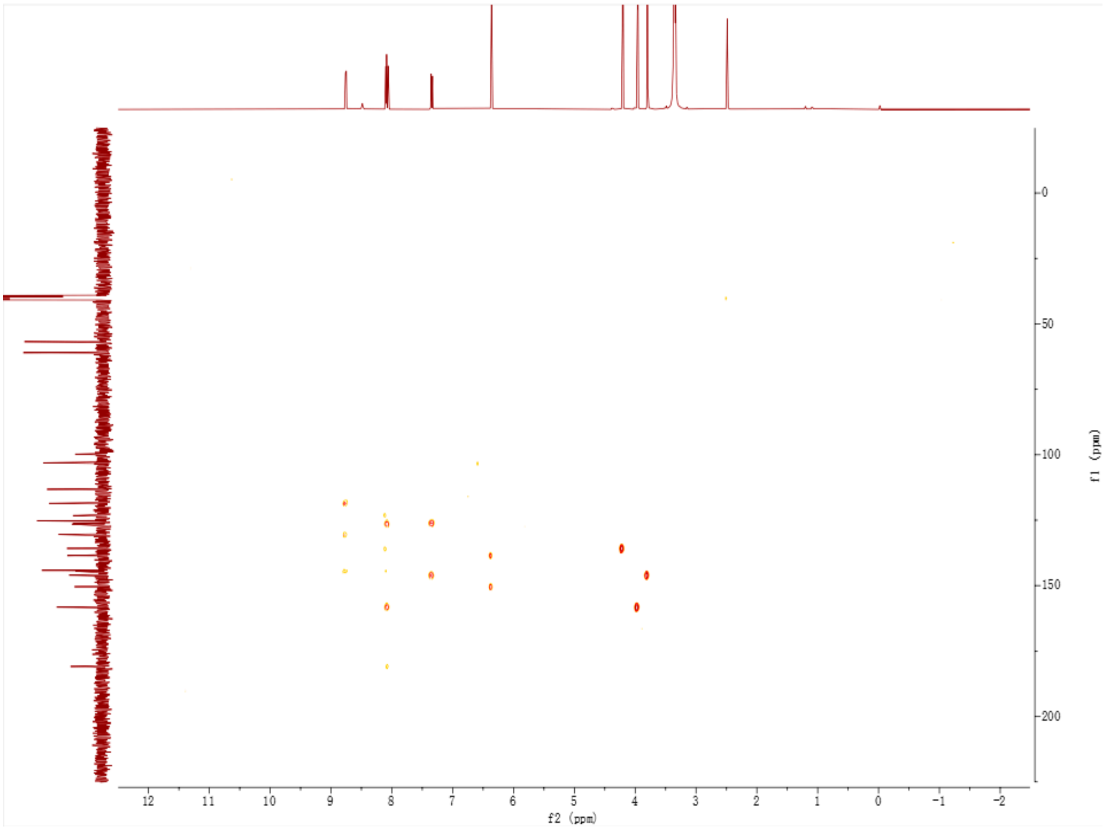

^{1}H-^{1}H COSY spectrum of Filiforidine

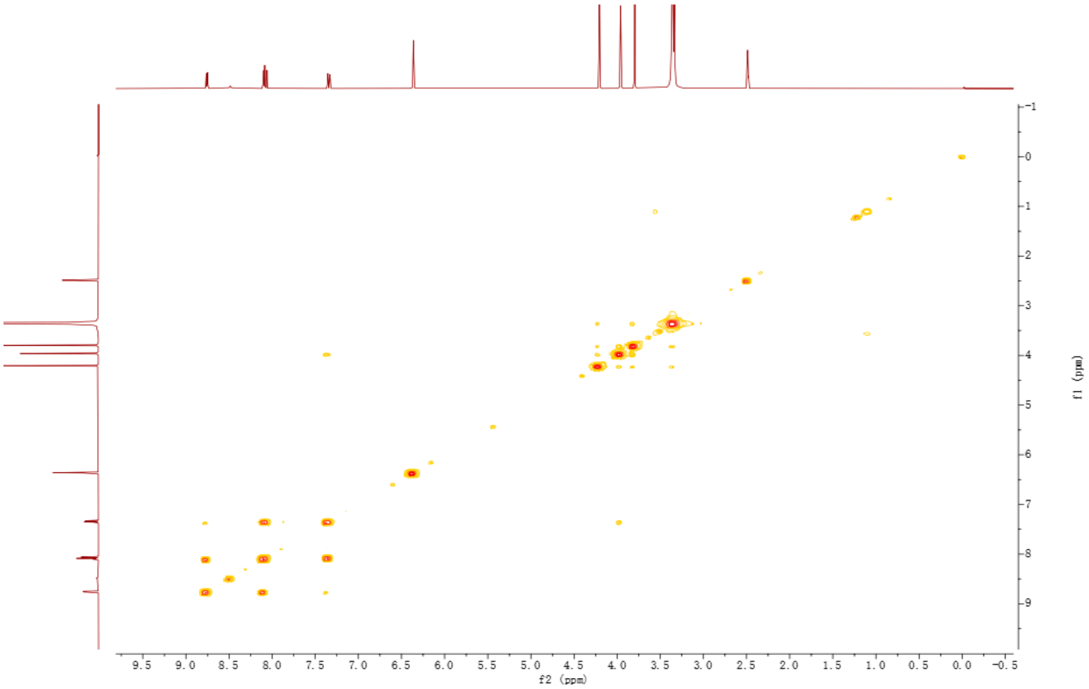

HPLC of Filiforidine

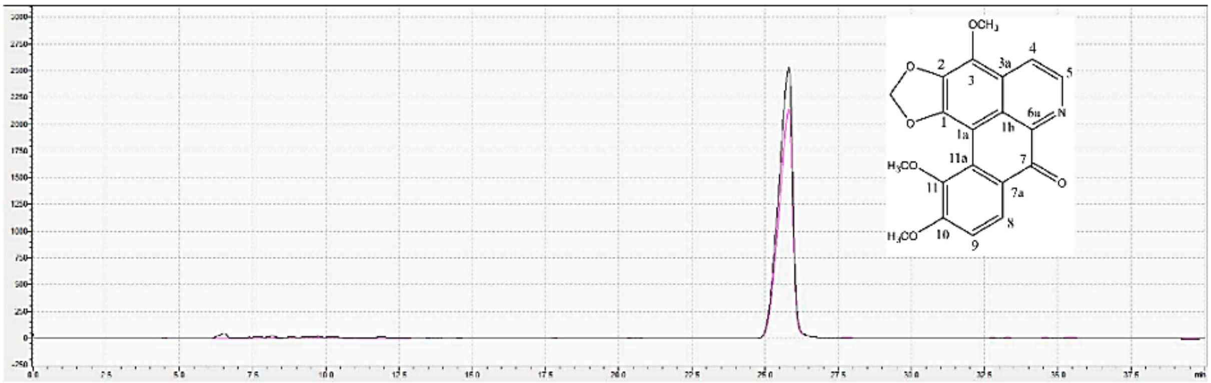

The UV spectrum of Filiforidine

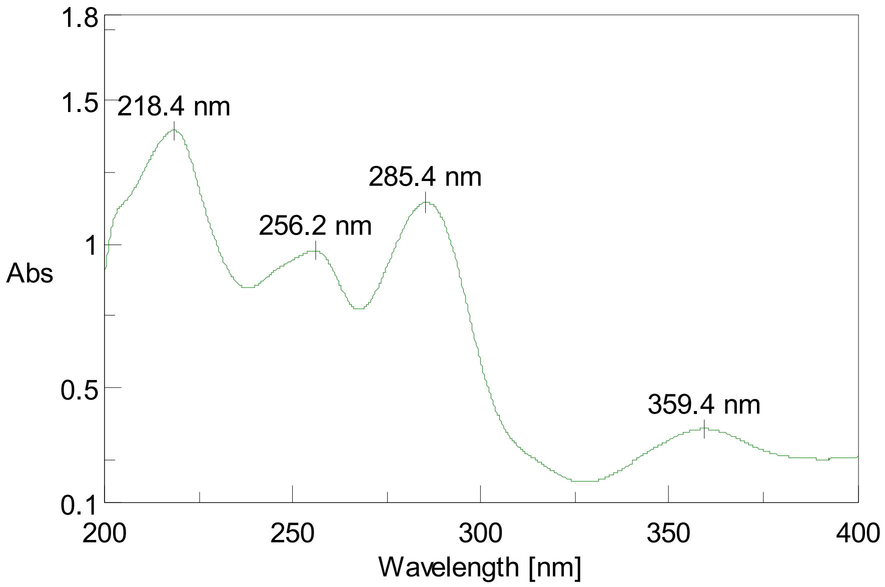

The IR spectrum of Filiforidine

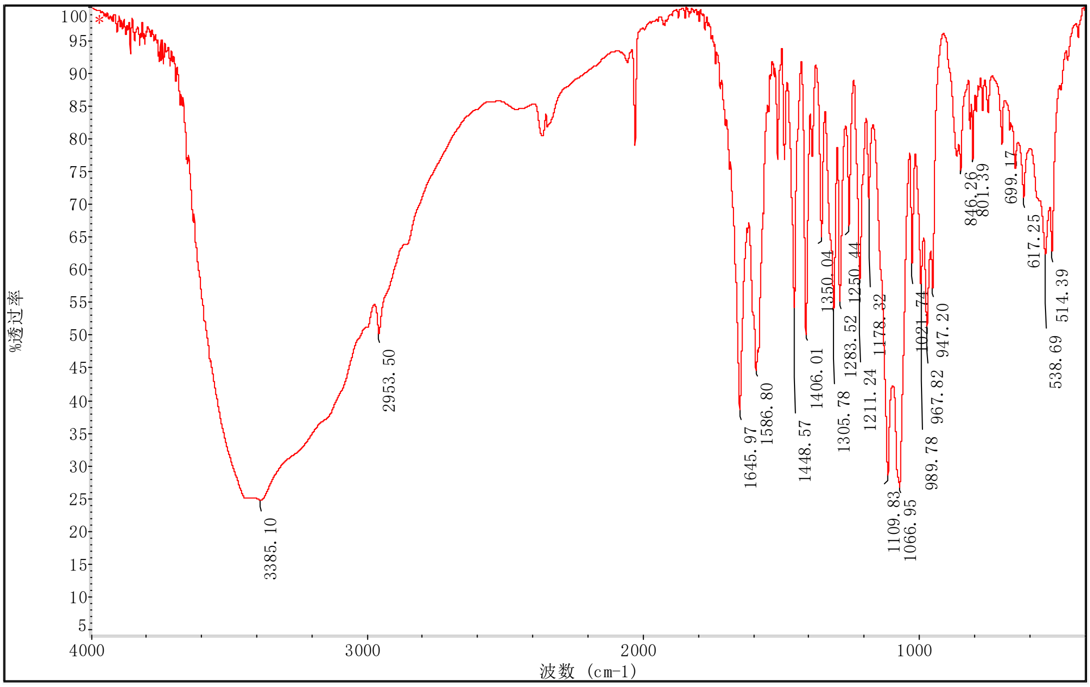

The mass spectrum of Filiforidine

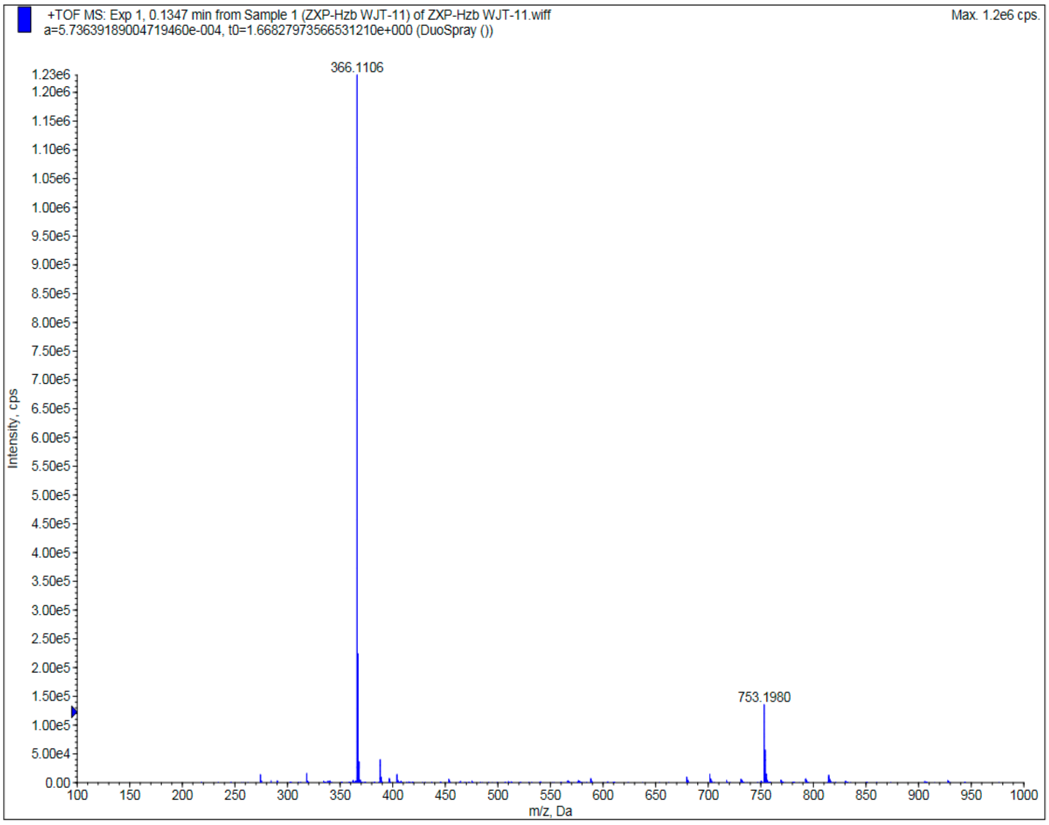

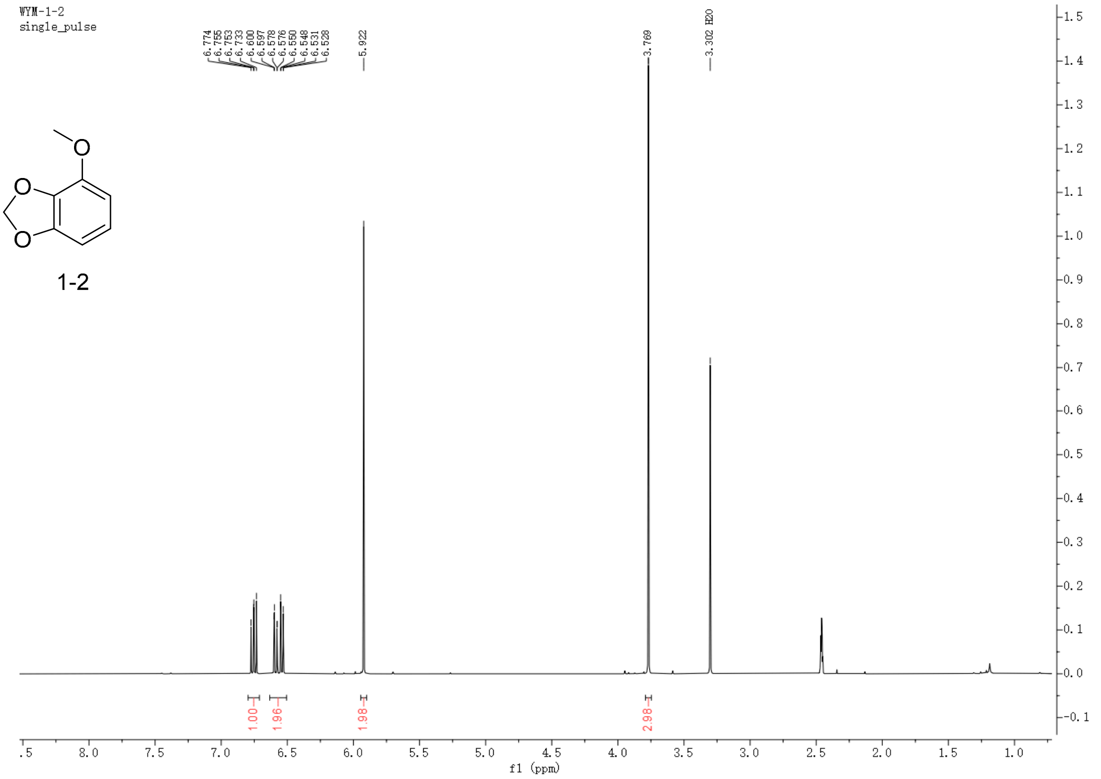
WYM-1-2
single_pulse
6.774
6.755
6.753
6.733
6.600
6.597
6.578
6.576
6.550
6.548
6.531
6.528
5.922
3.769
3.302 H2O
1.00
1.96
1.98
2.98
1-2
f1 (ppm)

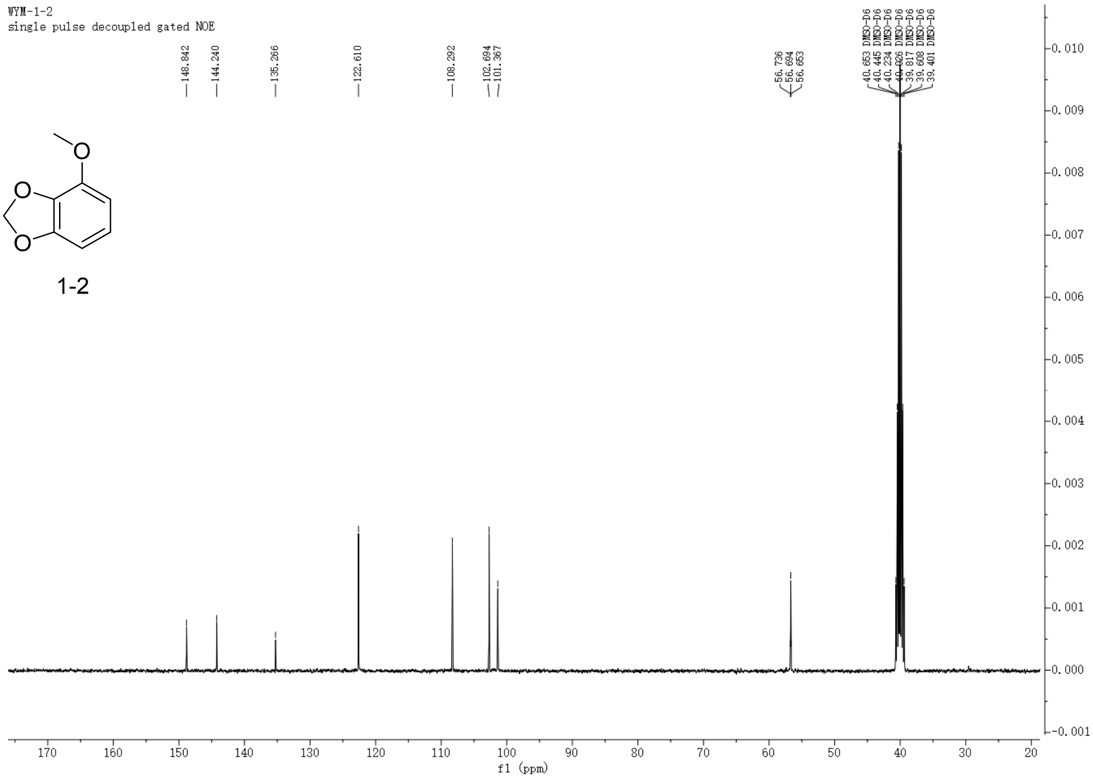
WYM-1-2
single pulse decoupled gated NOE
148.842
144.240
135.266
122.610
108.292
102.694
101.367
56.736
56.694
56.653
40.653 DMSO-D6
40.445 DMSO-D6
40.234 DMSO-D6
40.026 DMSO-D6
39.817 DMSO-D6
39.608 DMSO-D6
39.401 DMSO-D6
1-2
f1 (ppm)

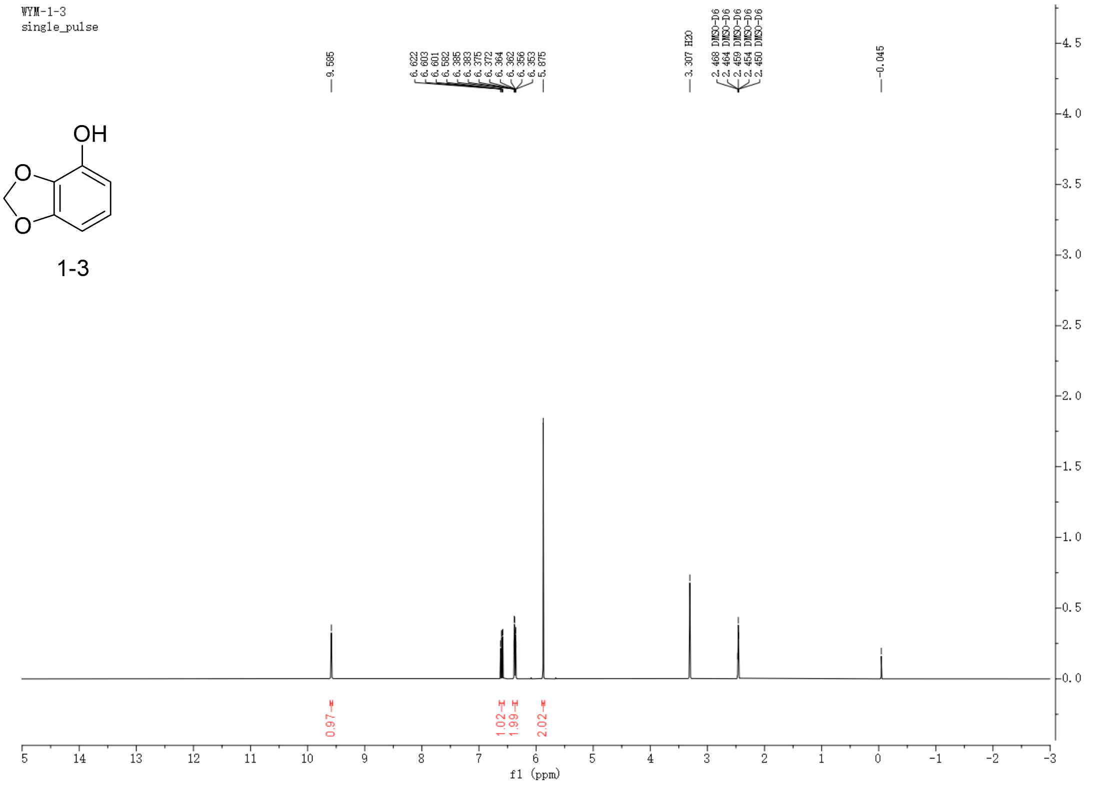
WYM-1-3
single_pulse
9.585
6.622
6.603
6.601
6.582
6.385
6.383
6.375
6.372
6.364
6.362
6.356
6.353
5.875
3.307 H2O
2.468 DMSO-D6
2.464 DMSO-D6
2.459 DMSO-D6
2.454 DMSO-D6
2.450 DMSO-D6
-0.045
OH
O
O
1-3
0.97
1.02
1.99
2.02
f1 (ppm)

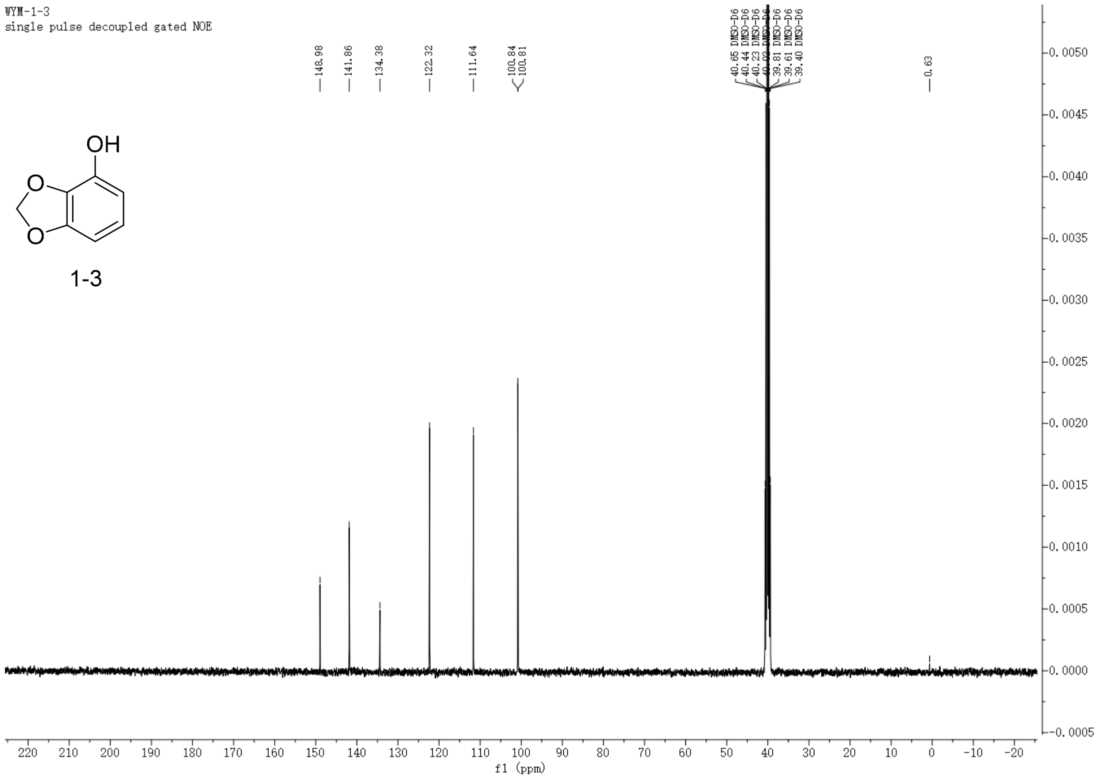
WYM-1-3
single pulse decoupled gated NOE
148.98
141.86
134.38
122.32
111.64
100.84
100.81
40.65 DMSO-D6
40.44 DMSO-D6
40.23 DMSO-D6
39.81 DMSO-D6
39.61 DMSO-D6
39.40 DMSO-D6
0.63
OH
O
O
1-3
f1 (ppm)

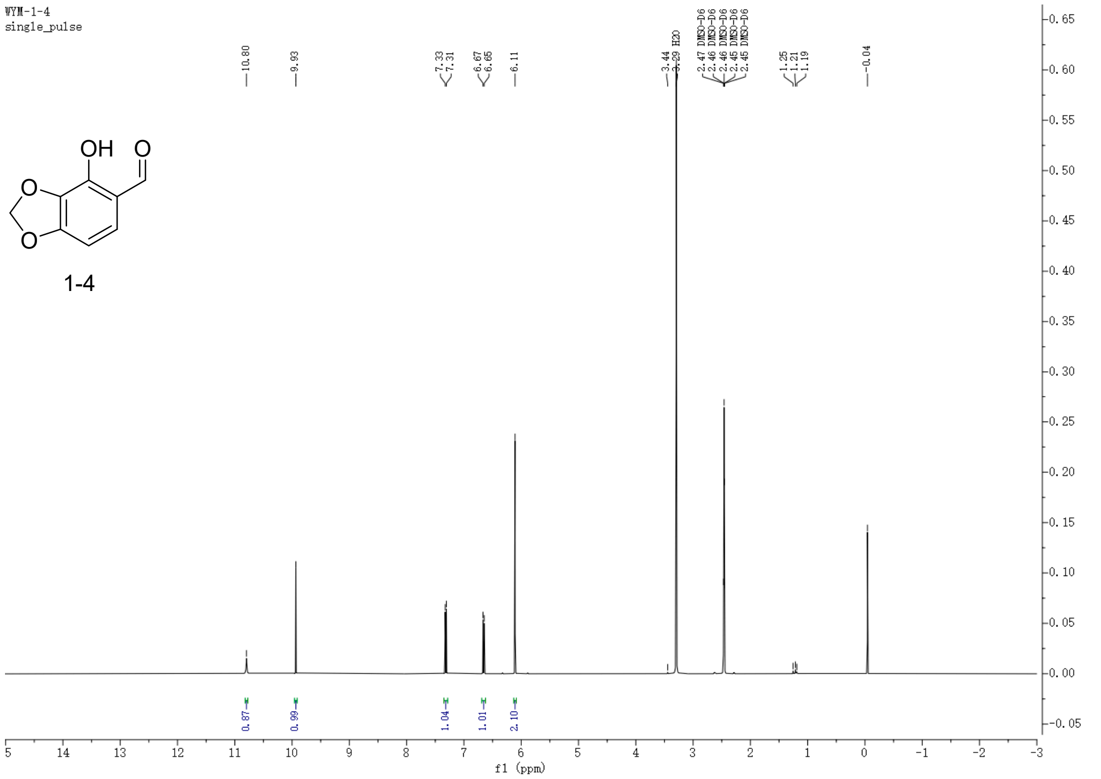
WYM-1-4
single_pulse
10.80
9.93
7.33
7.31
6.67
6.65
6.11
3.44
3.29 H2O
2.47 DMSO-D6
2.46 DMSO-D6
2.46 DMSO-D6
2.45 DMSO-D6
2.45 DMSO-D6
1.25
1.21
1.19
-0.04
OH
O
1-4
0.87
0.99
1.04
1.01
2.10
f1 (ppm)

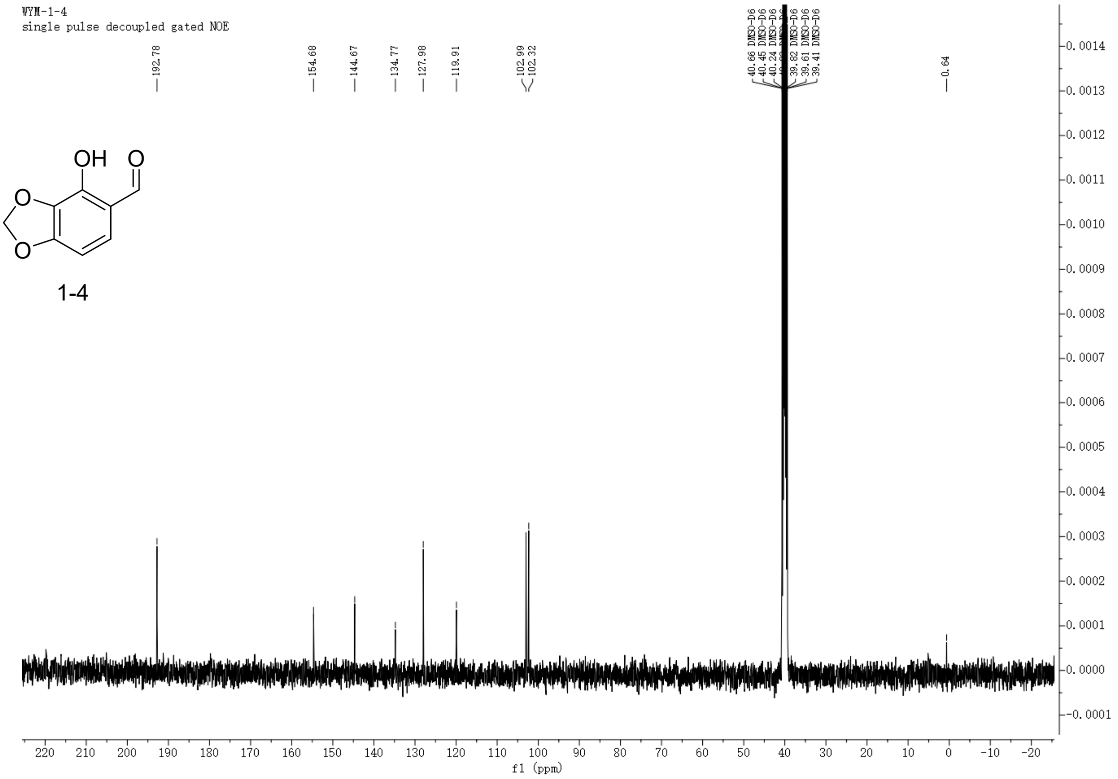
WYM-1-4
single pulse decoupled gated NOE
192.78
154.68
144.67
134.77
127.98
119.91
102.99
102.32
40.66 DMSO-D6
40.45 DMSO-D6
40.24 DMSO-D6
39.82 DMSO-D6
39.61 DMSO-D6
39.41 DMSO-D6
0.64
OH
O
1-4
f1 (ppm)

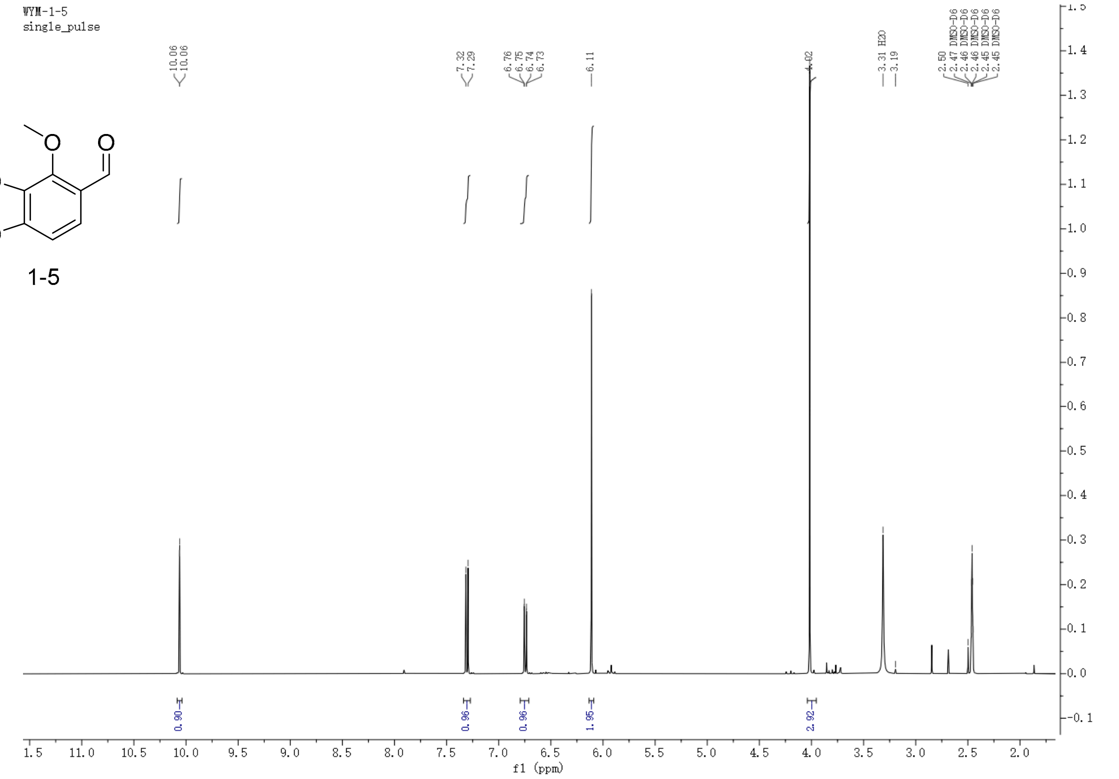
WYM-1-5
single_pulse
1-5
10.06
10.06
7.32
7.29
6.76
6.75
6.74
6.73
6.11
4.02
3.31 H2O
3.19
2.50
2.47 DMSO-D6
2.46 DMSO-D6
2.46 DMSO-D6
2.45 DMSO-D6
2.45 DMSO-D6
0.90
0.96
0.96
1.95
2.92
f1 (ppm)

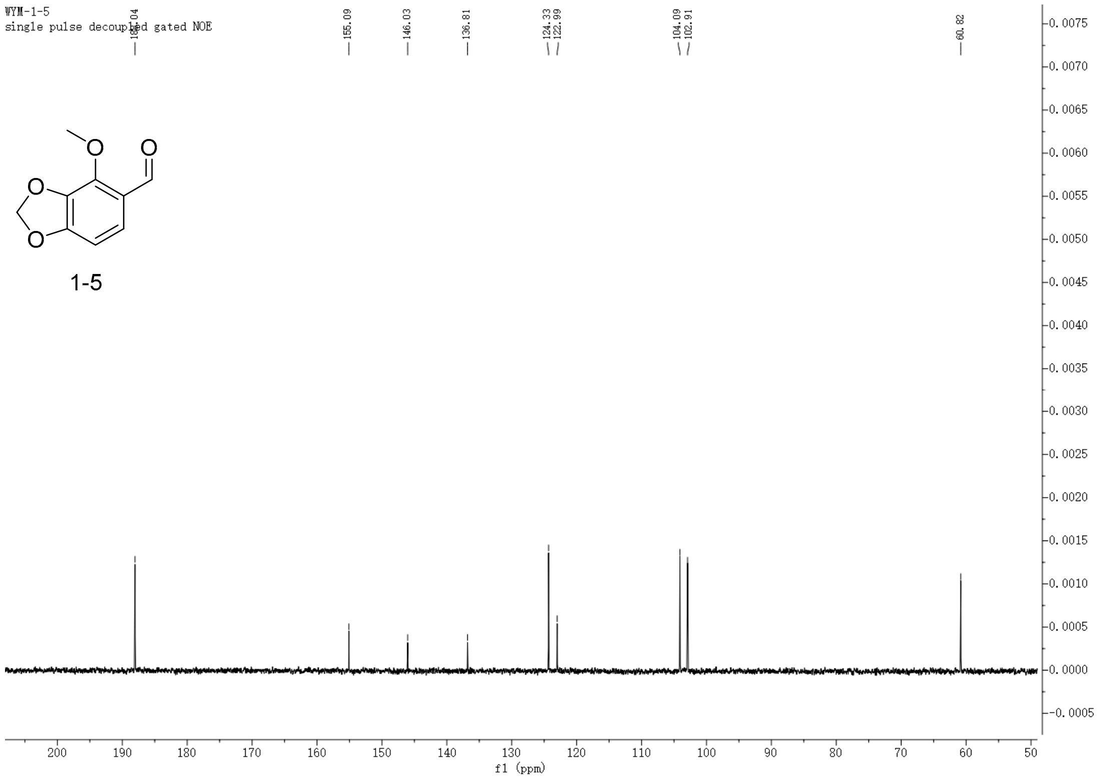
WYM-1-5
single pulse decoupled gated NOE
1-5
155.09
146.03
136.81
124.33
122.99
104.09
102.91
60.82
f1 (ppm)

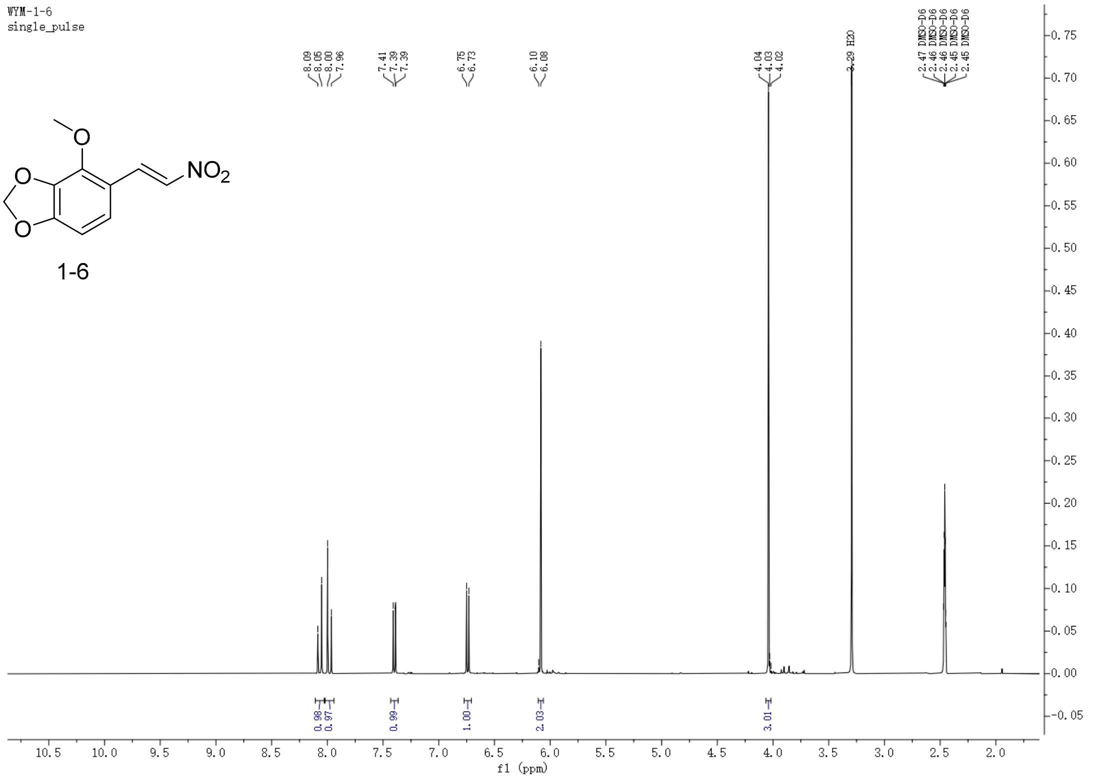
WYM-1-6
single_pulse
8.09 8.05 8.00 7.96
7.41 7.39 7.39
6.75 6.73
6.10 6.08
4.04 4.03 4.02
3.29 H2O
2.47 DMSO-D6 2.46 DMSO-D6 2.46 DMSO-D6 2.45 DMSO-D6 2.45 DMSO-D6
NO2
1-6
0.98 0.97 0.99 1.00 2.03 3.01
f1 (ppm)

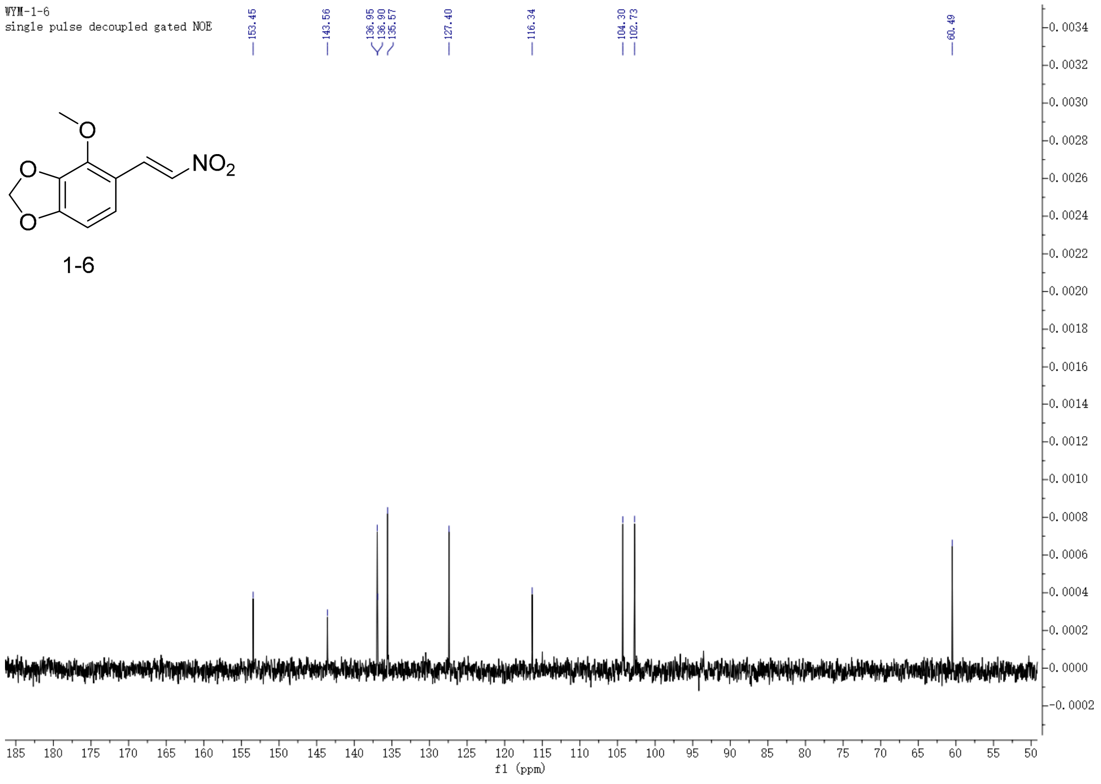
WYM-1-6
single pulse decoupled gated NOE
153.45
143.56
136.95 136.90 135.57
127.40
116.34
104.30 102.73
60.49
NO2
1-6
f1 (ppm)

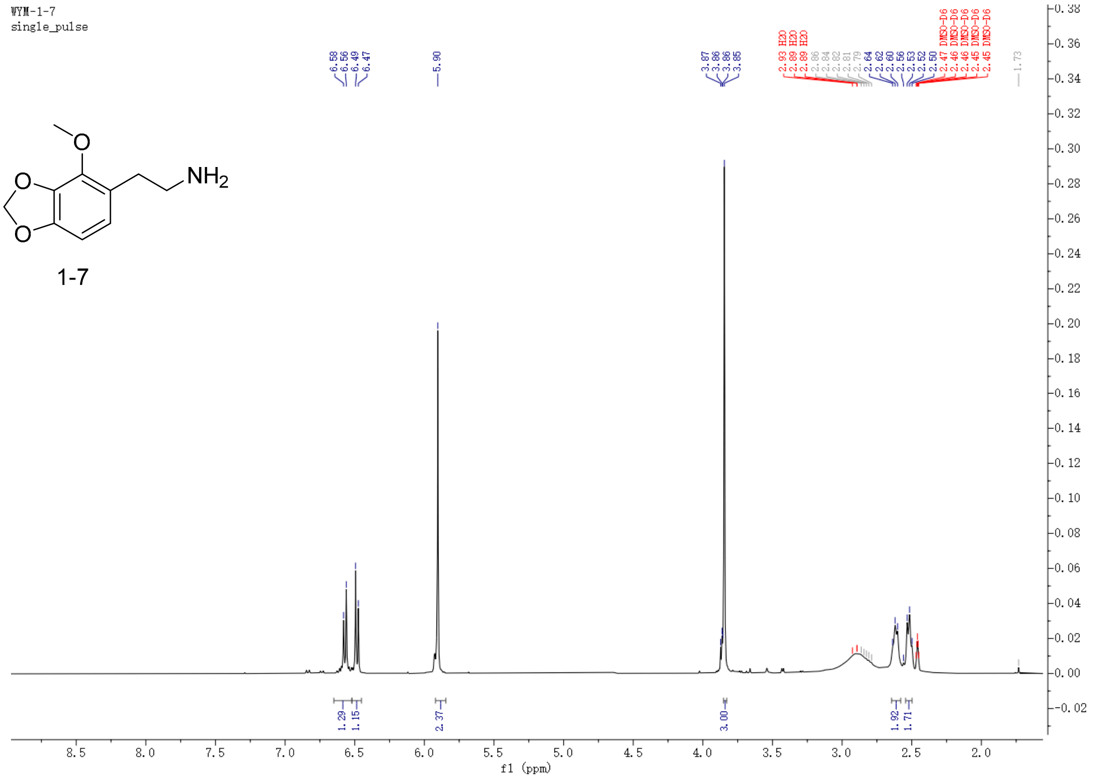
WYM-1-7
single_pulse
NH2
1-7
6.58
6.56
6.49
6.47
5.90
3.87
3.86
3.86
3.85
2.64
2.62
2.60
2.56
2.53
2.52
2.50
1.73
1.29
1.15
2.37
3.00
1.92
1.71
f1 (ppm)

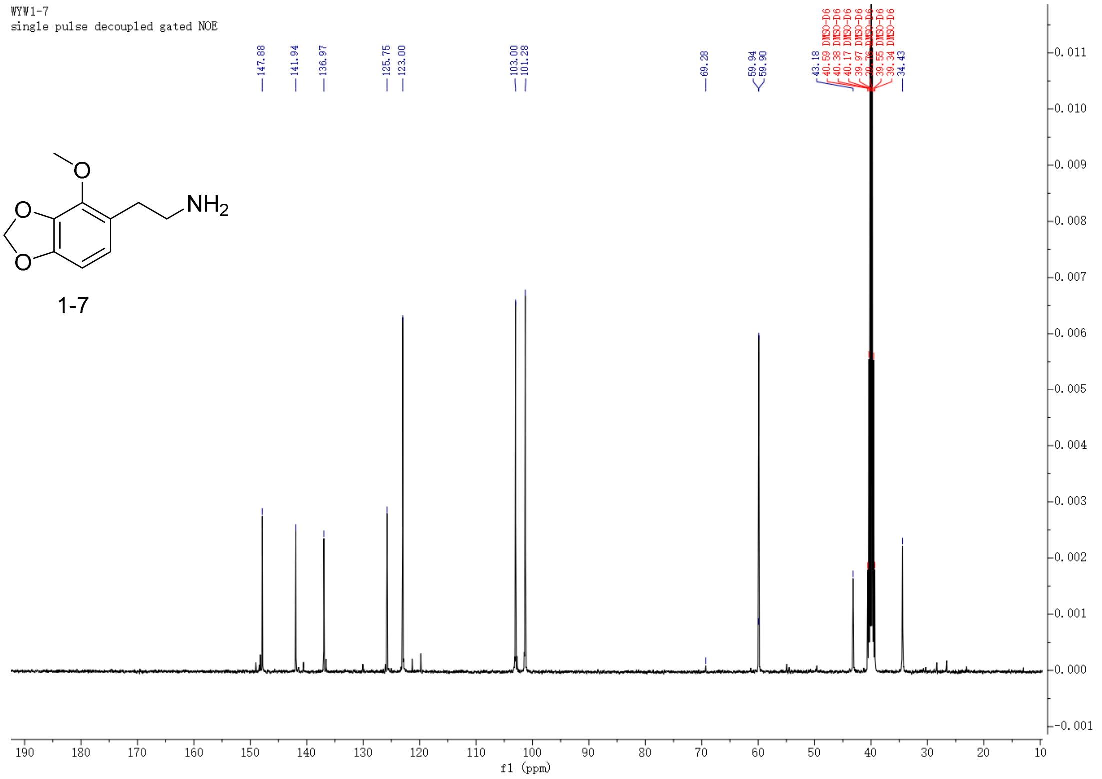
WYW1-7
single pulse decoupled gated NOE
NH2
1-7
147.88
141.94
136.97
125.75
123.00
103.00
101.28
69.28
59.94
59.90
43.18
34.43
f1 (ppm)

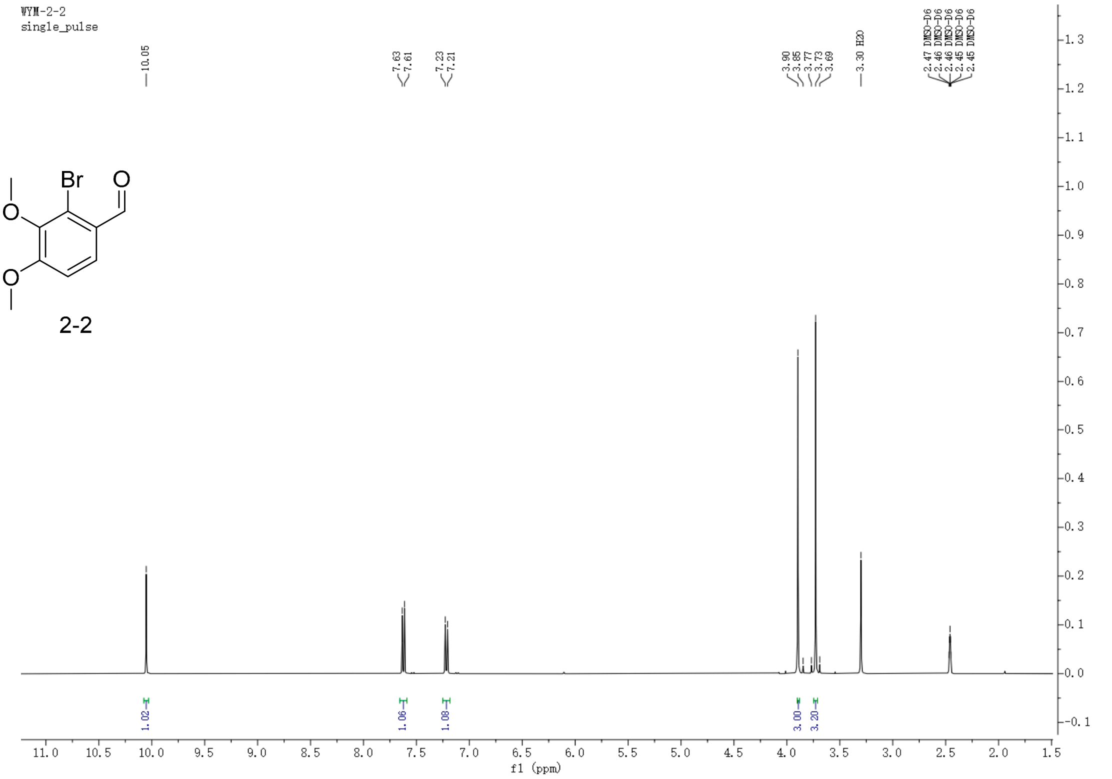
WYM-2-2
single_pulse
10.05
7.63
7.61
7.23
7.21
3.90
3.85
3.77
3.73
3.69
3.30 H2O
2.47 DMSO-D6
2.46 DMSO-D6
2.46 DMSO-D6
2.45 DMSO-D6
2.45 DMSO-D6
Br
O
O
O
2-2
1.02
1.06
1.08
3.00
3.20
f1 (ppm)

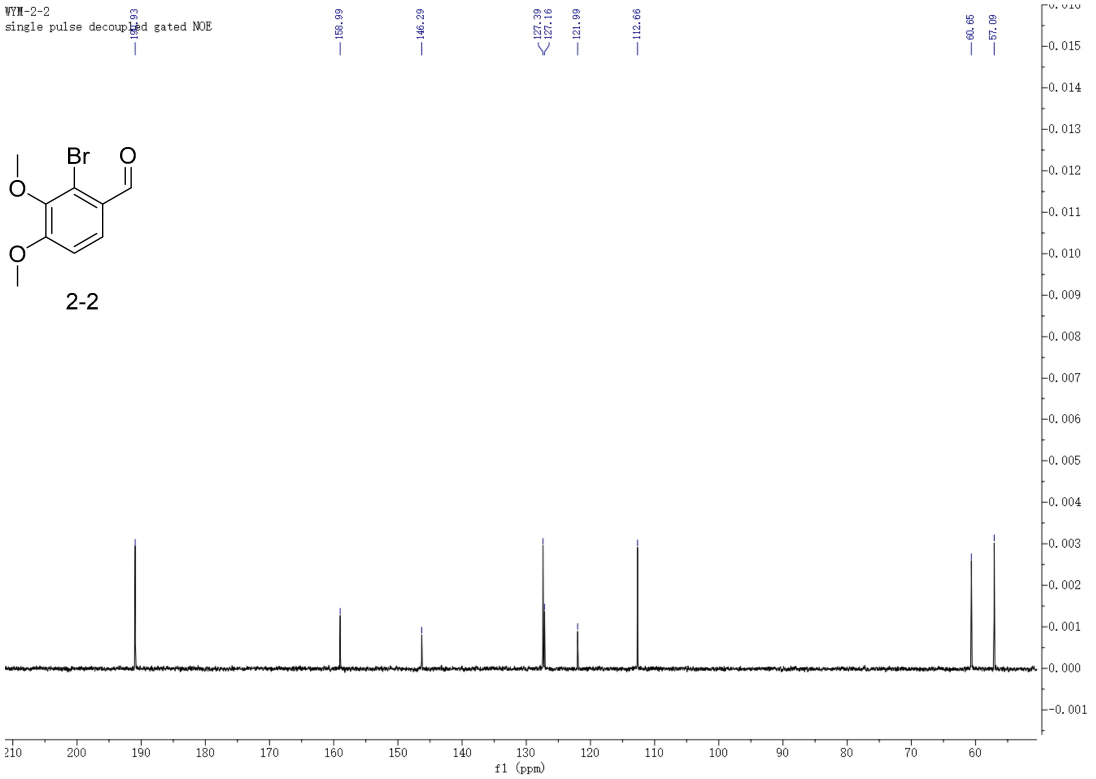
WYM-2-2
single pulse decoupled gated NOE
190.93
158.99
146.29
127.39
127.16
121.99
112.66
60.65
57.09
Br
O
O
O
2-2
f1 (ppm)

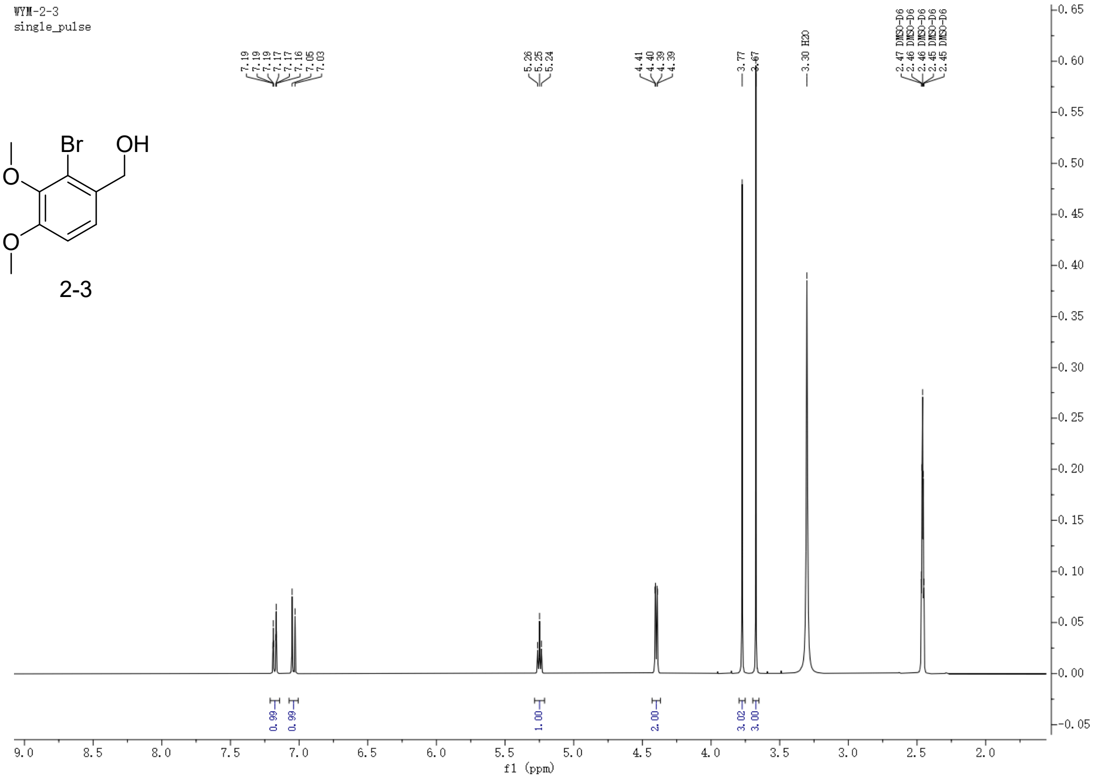
WYM-2-3
single_pulse
7.19
7.19
7.17
7.17
7.16
7.05
7.03
5.26
5.25
5.24
4.41
4.40
4.39
4.39
3.77
3.67
3.30 H2O
2.47 DMSO-D6
2.46 DMSO-D6
2.46 DMSO-D6
2.45 DMSO-D6
2.45 DMSO-D6
Br
OH
O
O
2-3
0.99
0.99
1.00
2.00
3.02
3.00
f1 (ppm)

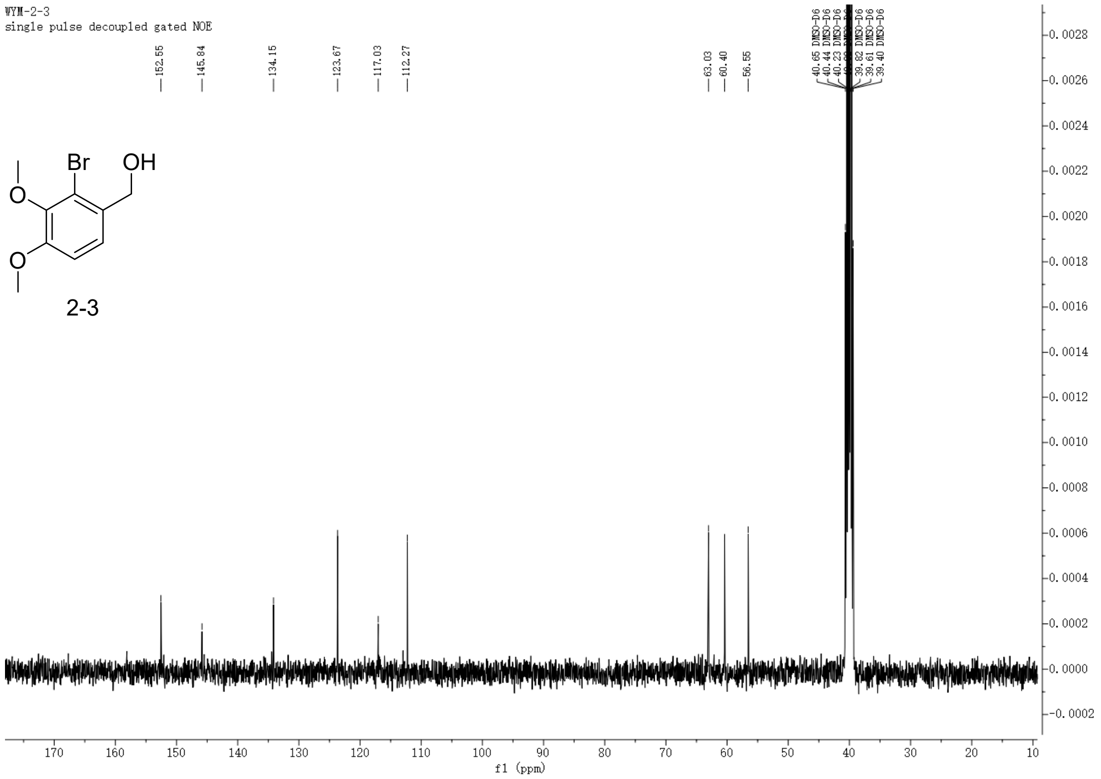
WYM-2-3
single pulse decoupled gated NOE
152.55
145.84
134.15
123.67
117.03
112.27
63.03
60.40
56.55
40.65 DMSO-D6
40.44 DMSO-D6
40.23 DMSO-D6
39.82 DMSO-D6
39.61 DMSO-D6
39.40 DMSO-D6
Br
OH
O
O
2-3
f1 (ppm)

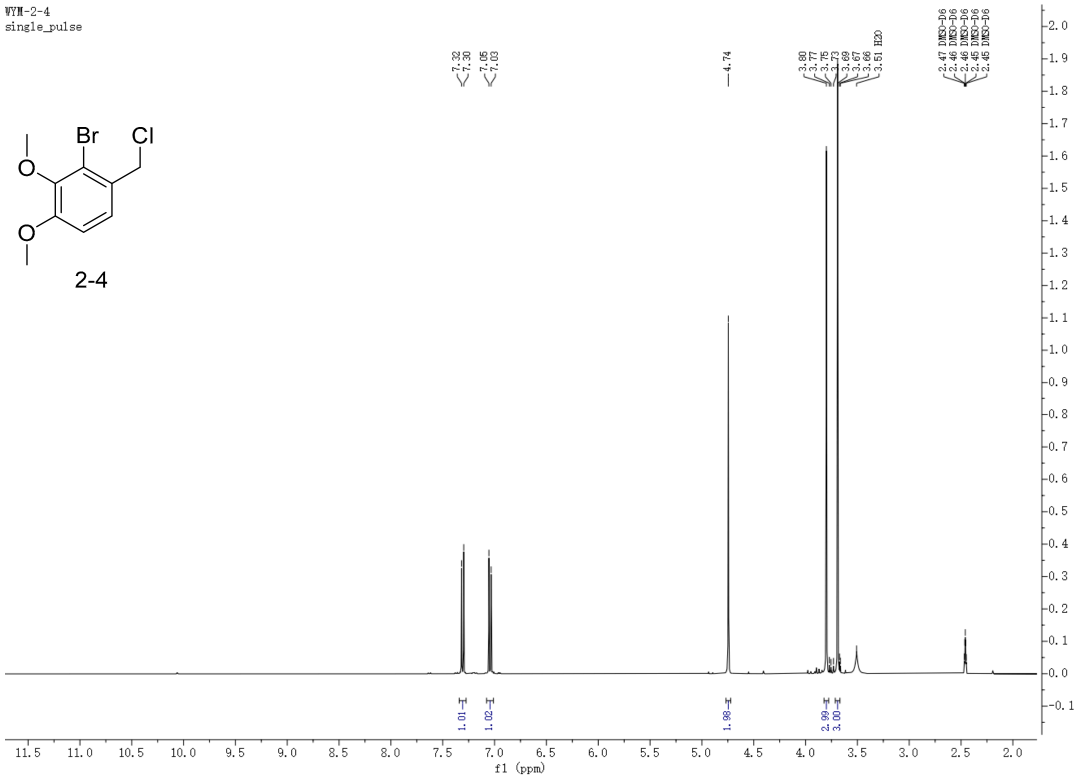
WYM-2-4
single_pulse
7.32
7.30
7.05
7.03
4.74
3.80
3.77
3.75
3.73
3.69
3.67
3.66
3.51 H2O
2.47 DMSO-D6
2.46 DMSO-D6
2.46 DMSO-D6
2.45 DMSO-D6
2.45 DMSO-D6
Br
Cl
O
O
2-4
1.01
1.02
1.98
2.99
3.00
f1 (ppm)

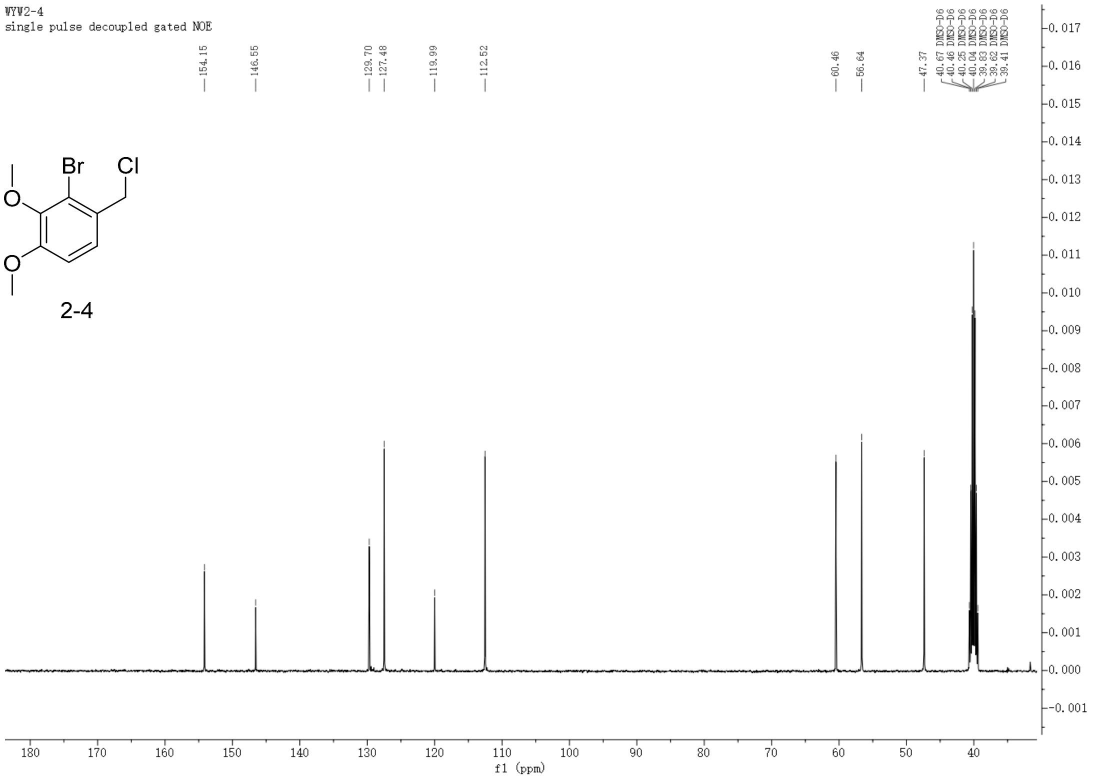
WYW2-4
single pulse decoupled gated NOE
154.15
146.55
129.70
127.48
119.99
112.52
60.46
56.64
47.37
40.67 DMSO-D6
40.46 DMSO-D6
40.25 DMSO-D6
40.04 DMSO-D6
39.83 DMSO-D6
39.62 DMSO-D6
39.41 DMSO-D6
Br
Cl
O
O
2-4
f1 (ppm)

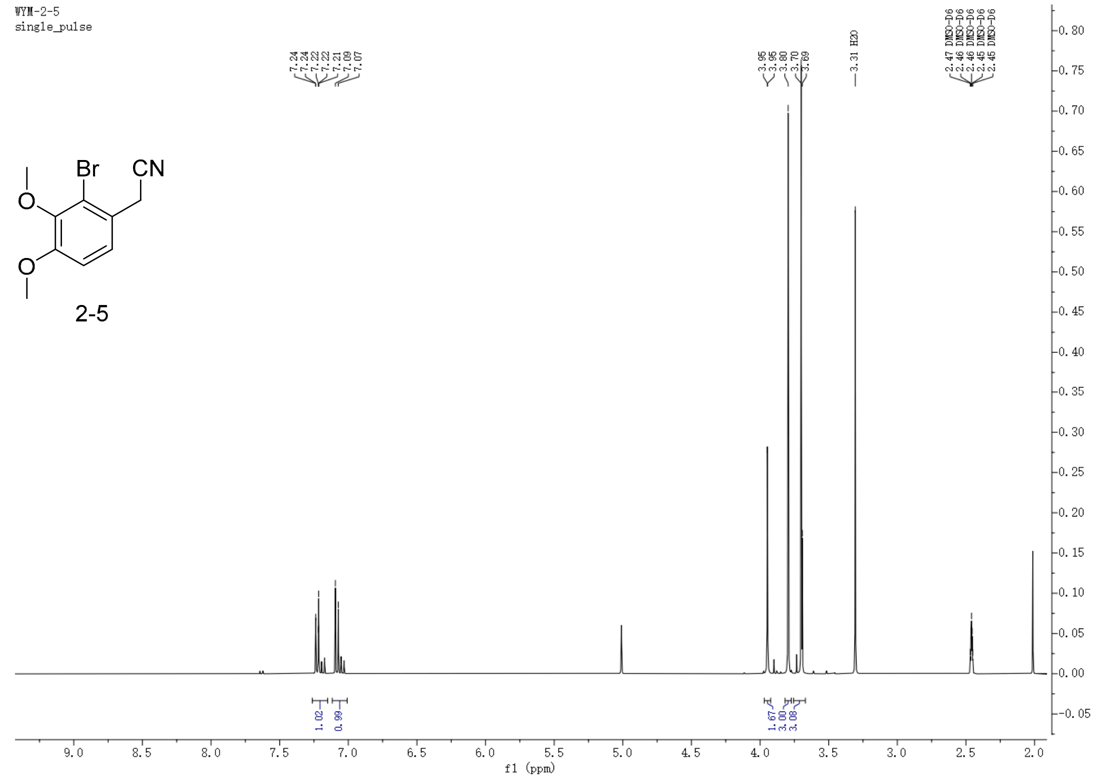
WYM-2-5
single_pulse
7.24 7.24 7.22 7.22 7.21 7.09 7.07
3.95 3.95 3.80 3.70 3.69
3.31 H2O
2.47 DMSO-D6 2.46 DMSO-D6 2.46 DMSO-D6 2.45 DMSO-D6 2.45 DMSO-D6
Br
CN
O
O
2-5
1.02
0.99
1.67
3.00
3.08
f1 (ppm)

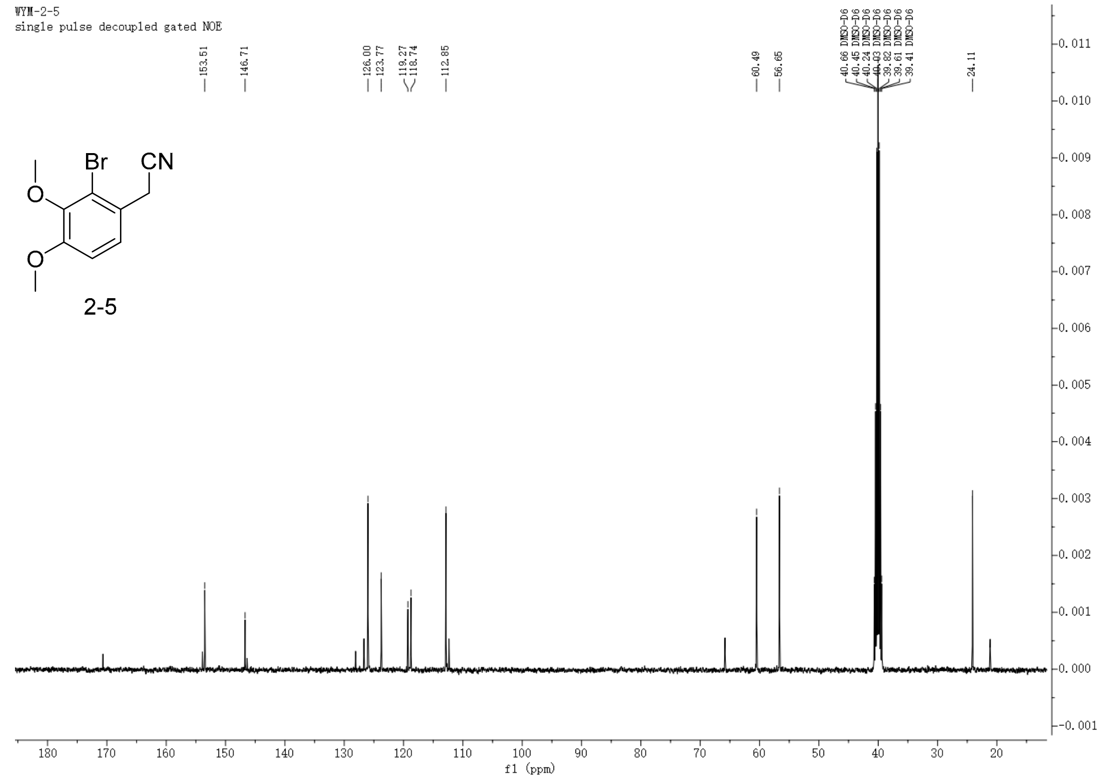
WYM-2-5
single pulse decoupled gated NOE
153.51
146.71
126.00 123.77 119.27 118.74
112.85
60.49
56.65
40.66 DMSO-D6 40.45 DMSO-D6 40.24 DMSO-D6 40.03 DMSO-D6 39.82 DMSO-D6 39.61 DMSO-D6 39.41 DMSO-D6
24.11
Br
CN
O
O
2-5
f1 (ppm)

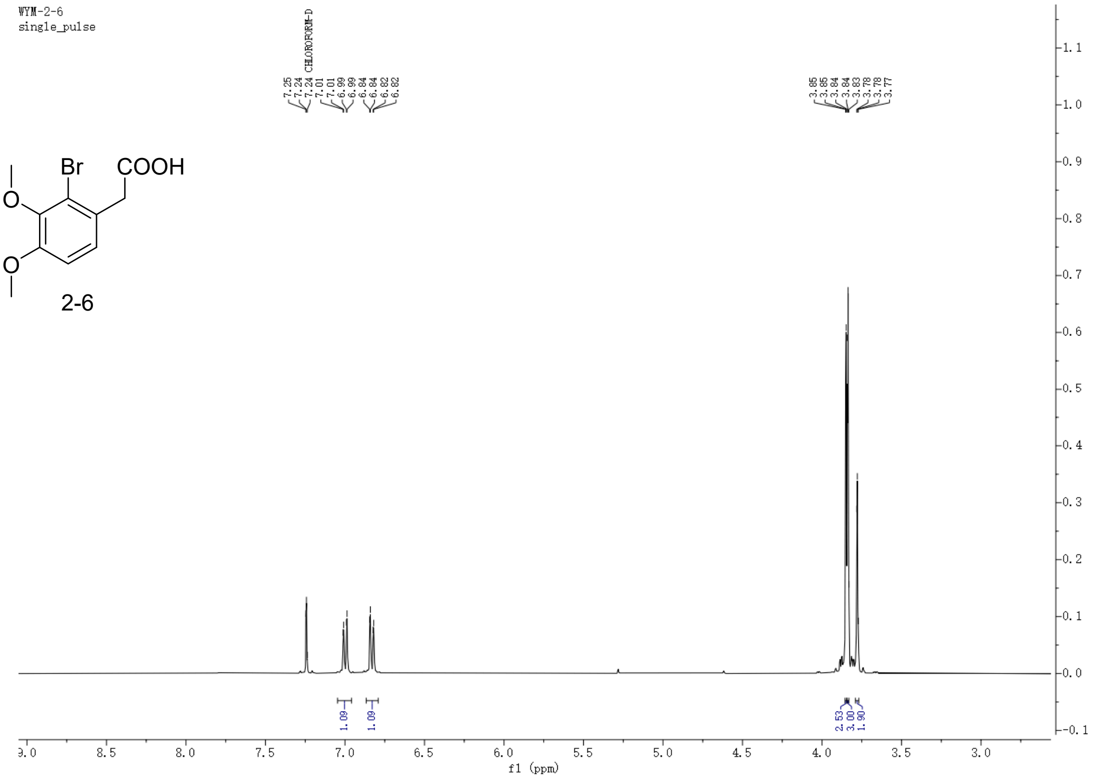
WYM-2-6
single_pulse
7.25
7.24
7.24 CHLOROFORM-D
7.01
7.01
6.99
6.99
6.84
6.84
6.82
6.82
3.85
3.85
3.84
3.84
3.83
3.78
3.77
Br
COOH
O
O
2-6
1.09
1.09
2.53
3.00
1.90
f1 (ppm)

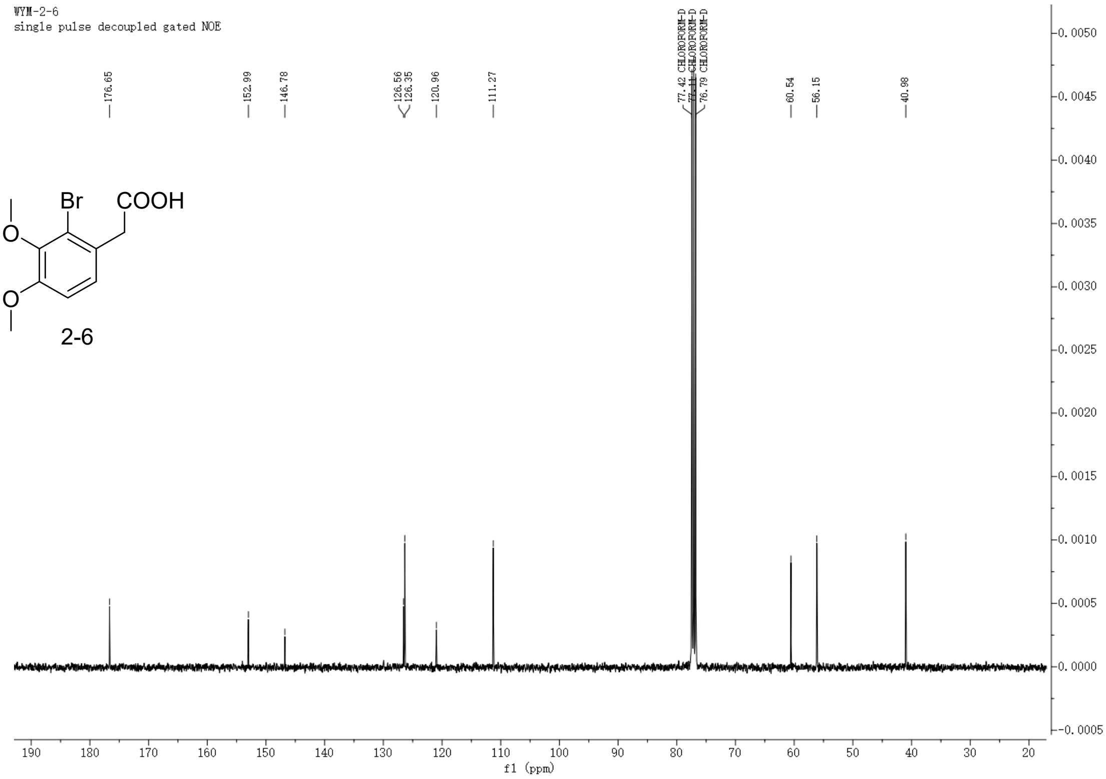
WYM-2-6
single pulse decoupled gated NOE
176.65
152.99
146.78
126.56
126.35
120.96
111.27
77.42 CHLOROFORM-D
76.79 CHLOROFORM-D
60.54
56.15
40.98
Br
COOH
O
O
2-6
f1 (ppm)

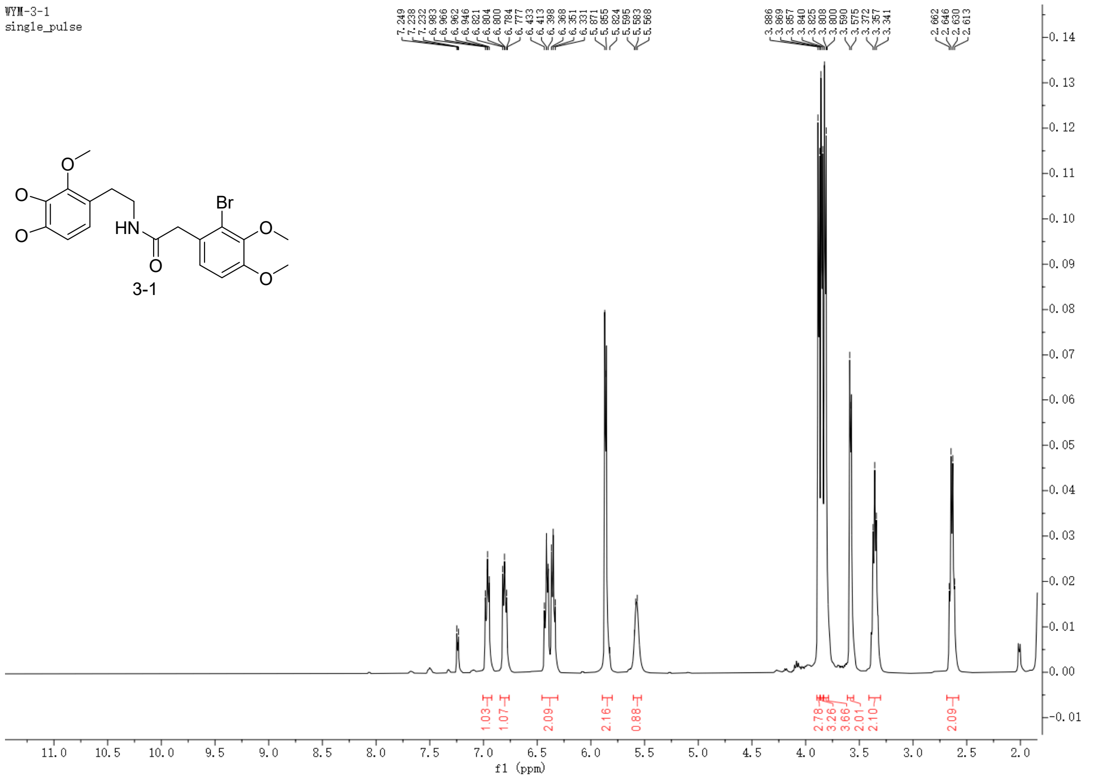
WYM-3-1
single_pulse
7.249 7.238 7.232 6.983 6.966 6.962 6.946 6.821 6.804 6.800 6.784 6.777 6.433 6.413 6.398 6.368 6.351 6.331 5.871 5.855 5.624 5.595 5.583 5.568
3.886 3.869 3.857 3.840 3.825 3.808 3.800 3.590 3.575 3.372 3.357 3.341
2.662 2.646 2.630 2.613
Br
HN
O
3-1
1.03 1.07 2.09 2.16 0.88 2.78 3.26 3.66 2.01 2.10 2.09
f1 (ppm)

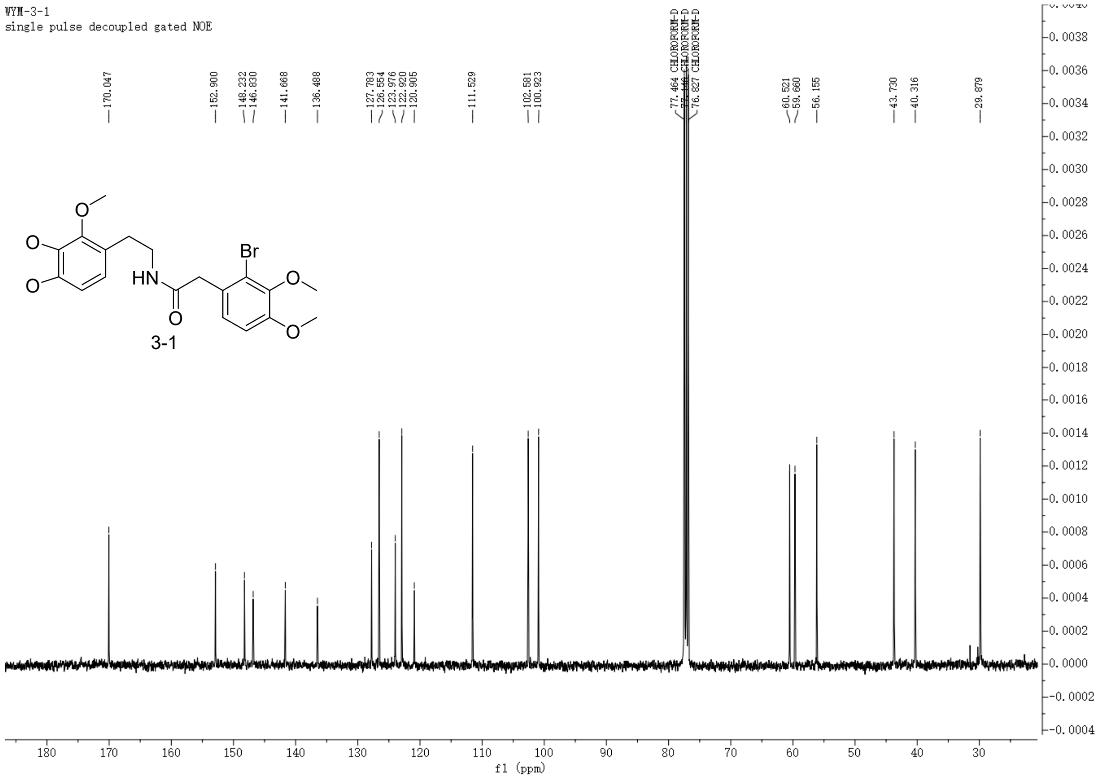
WYM-3-1
single pulse decoupled gated NOE
170.047
152.900 148.232 146.830 141.668 136.488
127.783 126.554 123.976 122.920 120.905
111.529
102.581 100.923
77.464 CHLOROFORM-D
[illegible] CHLOROFORM-D
76.827 CHLOROFORM-D
60.521 59.660 56.155
43.730 40.316
29.879
Br
HN
O
3-1
f1 (ppm)

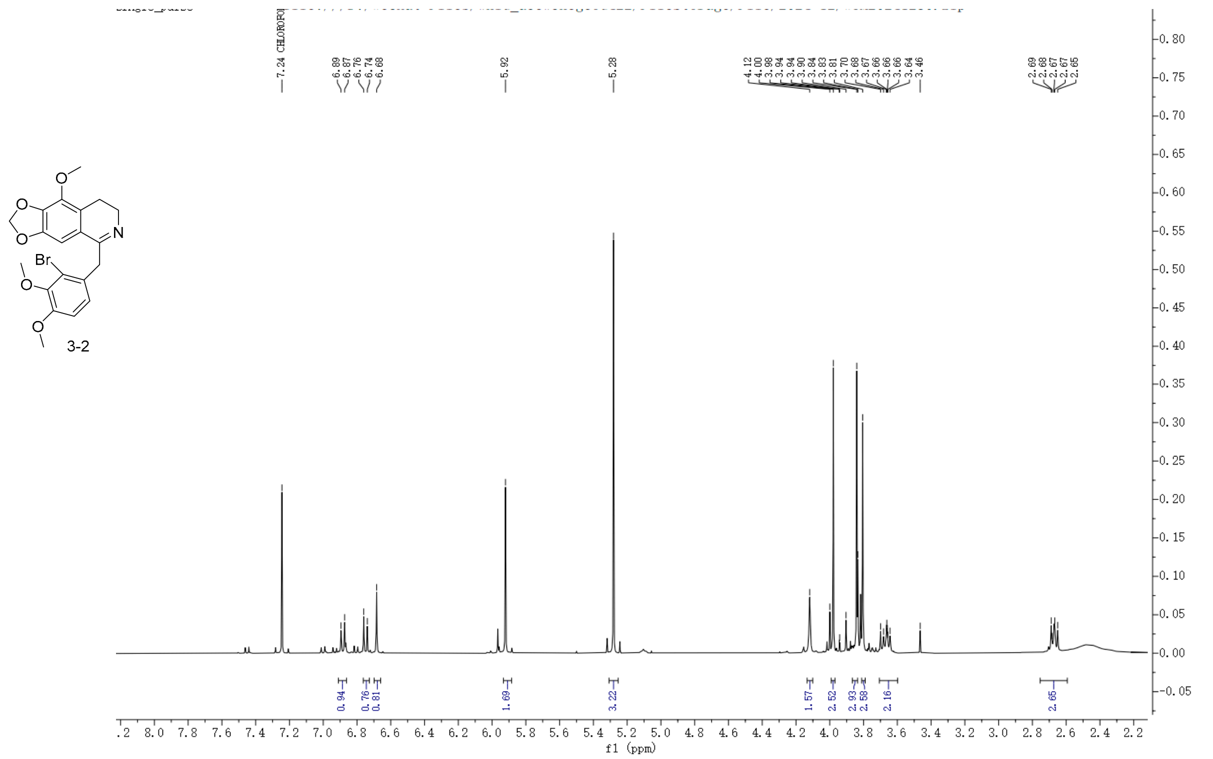

7.24 CHLOROFORM
6.89
6.87
6.76
6.74
6.68
5.92
5.28
4.12
4.00
3.98
3.94
3.94
3.90
3.84
3.83
3.81
3.70
3.68
3.67
3.66
3.66
3.65
3.64
3.46
2.69
2.68
2.67
2.67
2.65
0.94
0.76
0.81
1.69
3.22
1.57
2.52
2.93
2.58
2.16
2.65
Br
N
O
3-2
f1 (ppm)

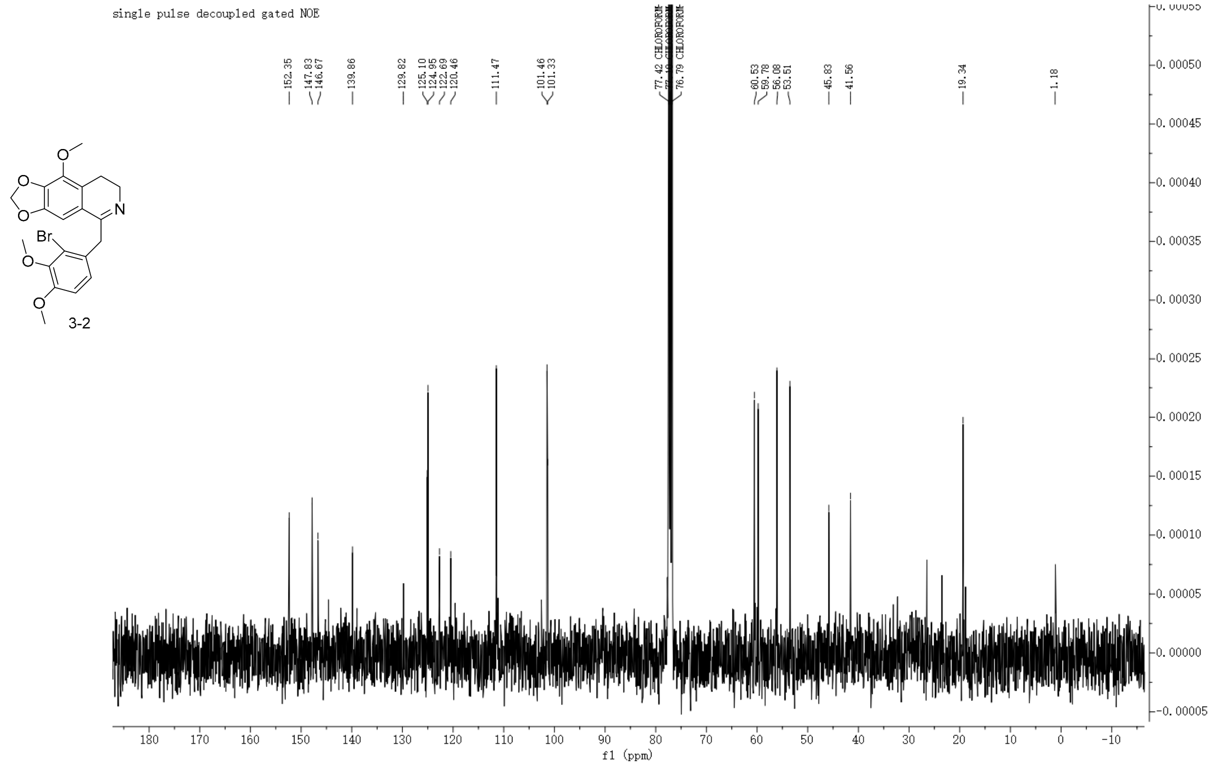

single pulse decoupled gated NOE
152.35
147.83
146.67
139.86
129.82
125.10
124.95
122.69
120.46
111.47
101.46
101.33
77.42 CHLOROFORM
76.79 CHLOROFORM
60.53
59.78
56.08
53.51
45.83
41.56
19.34
1.18
Br
N
O
3-2
f1 (ppm)

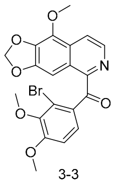
O
O
O
N
Br
O
O
O

3-3

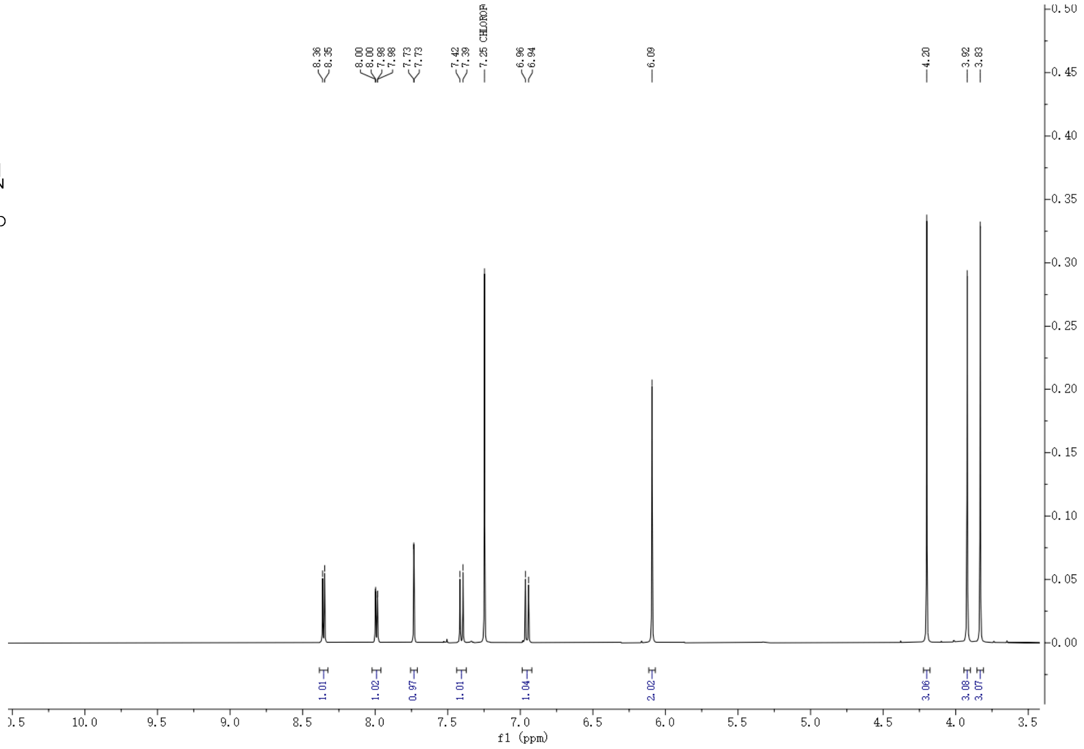
8.36
8.36
8.00
8.00
7.98
7.98
7.73
7.73
7.42
7.39
7.25 CHLOROF
6.96
6.94
6.09
4.20
3.92
3.83
1.01
1.02
0.97
1.01
1.04
2.02
3.06
3.08
3.07
f1 (ppm)

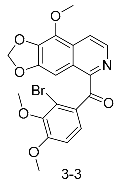
O
O
O
N
Br
O
O
O

3-3

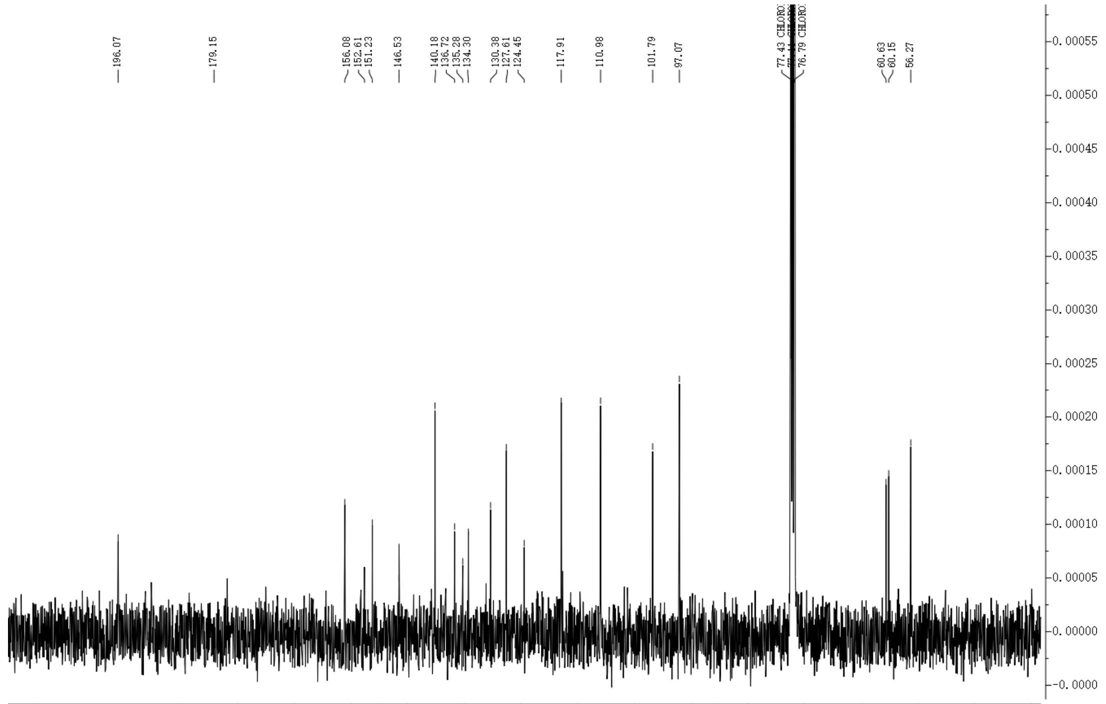
196.07
179.15
156.08
152.61
151.23
146.53
140.18
136.72
135.28
134.30
130.38
127.61
124.45
117.91
110.98
101.79
97.07
77.43 CHLORO
76.79 CHLORO
60.63
60.15
56.27
f1 (ppm)

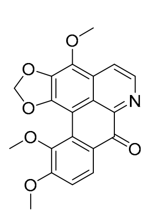

3-4

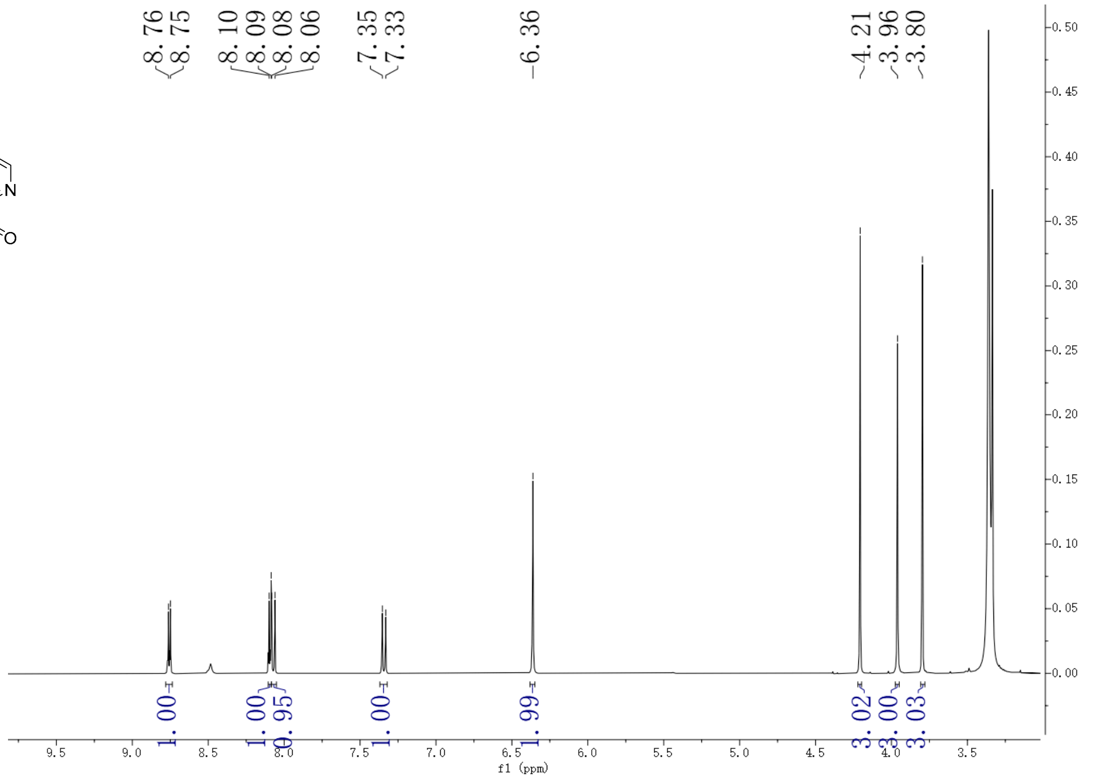
8.76
8.75
8.10
8.09
8.08
8.06
7.35
7.33
6.36
4.21
3.96
3.80
1.00
1.00
0.95
1.00
1.99
3.02
3.00
3.03
f1 (ppm)

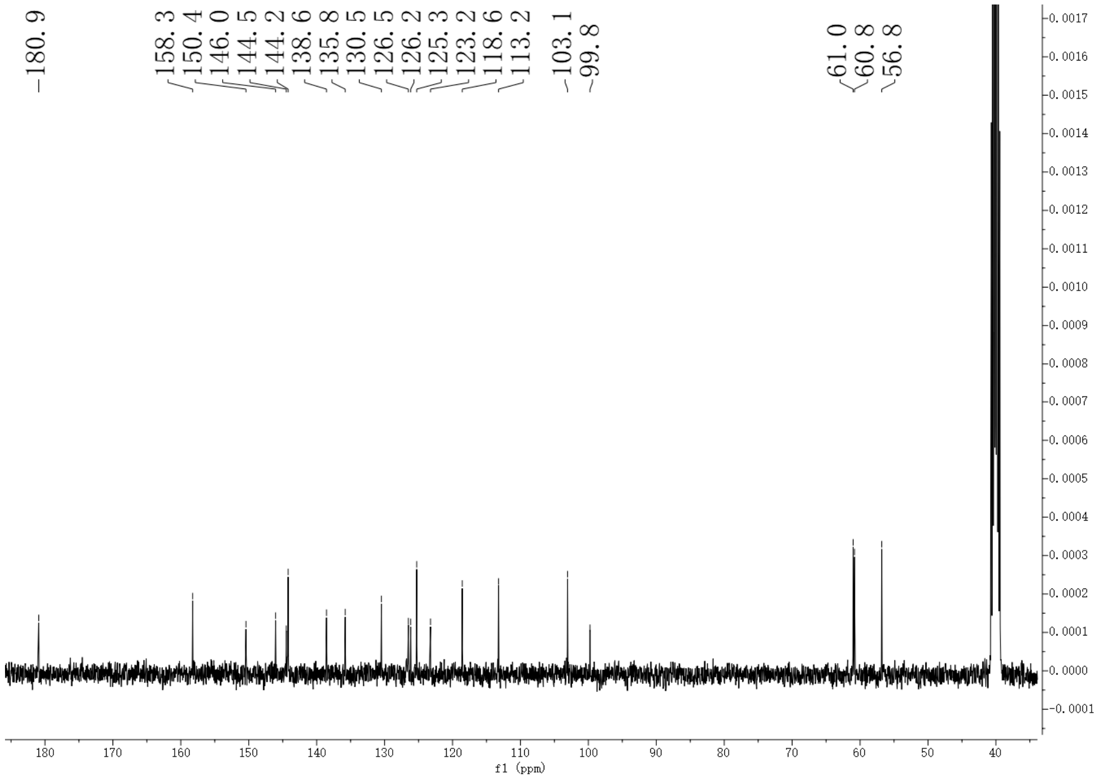
180.9
158.3
150.4
146.0
144.5
144.2
138.6
135.8
130.5
126.5
126.2
125.3
123.2
118.6
113.2
103.1
99.8
61.0
60.8
56.8
f1 (ppm)
